# Supplementary material for: Interfacing nickel nitride and nickel boosts both electrocatalytic hydrogen evolution and oxidation reactions
Source: Nat Commun. 2018 Oct 31;9:4531. doi: 10.1038/s41467-018-06728-7 (PMC6208398; doi:10.1038/s41467-018-06728-7)
Supplement: Supplementary file 1 — Supplementary Information [file 41467_2018_6728_MOESM1_ESM.pdf]

Supplementary Information

Interfacing nickel nitride and nickel boosts both electrocatalytic hydrogen evolution and oxidation reactions

Fuzhan Song,[1+] Wei Li,[1+] Jiaqi Yang,[2] Guanqun Han,[1] Peilin Liao,[2*] Yujie Sun[1,3*]

[1]Department of Chemistry & Biochemistry, Utah State University, Logan, UT 84322, United States

[2]School of Materials Engineering, Purdue University, West Lafayette, IN 47907, United States

[3]Department of Chemistry, University of Cincinnati, Cincinnati, OH 45221, United States

[+]These authors contributed equally: Fuzhan Song and Wei Li.

Correspondence and requests for materials should be addressed to Yujie Sun (email: yujie.sun@uc.edu) or Peilin Liao (email: lpl@purdue.edu).

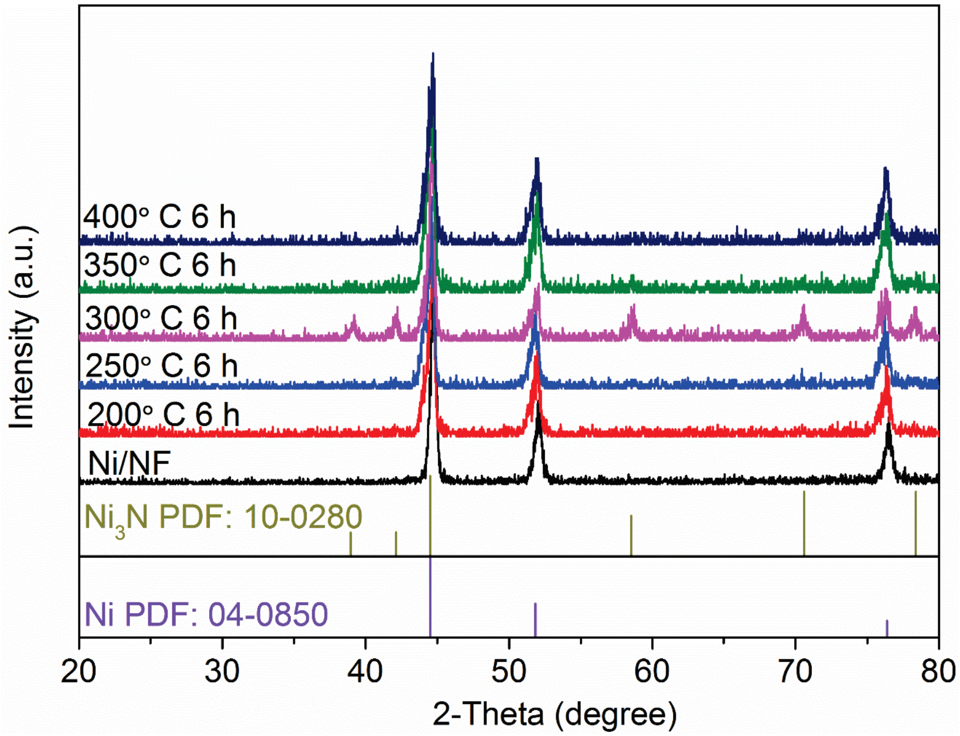

Supplementary Fig. 1. XRD patterns of Ni_3N/Ni/NF synthesized at different nitridation temperatures for 6 h.

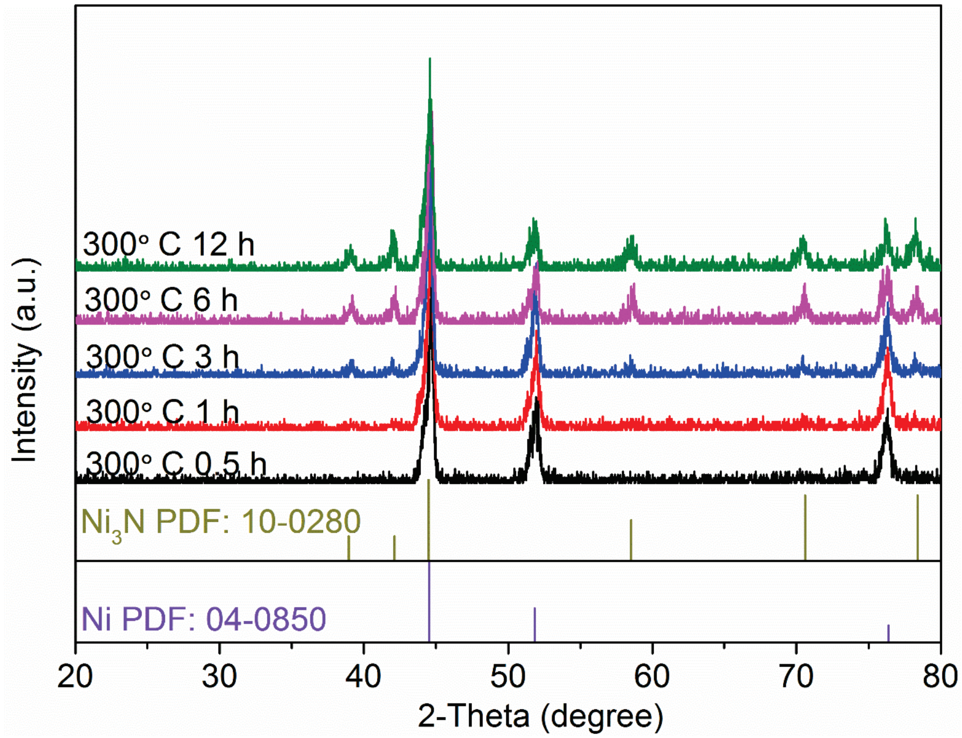

Supplementary Fig. 2. XRD patterns of Ni_3N/Ni/NF synthesized at 300 °C for different duration.

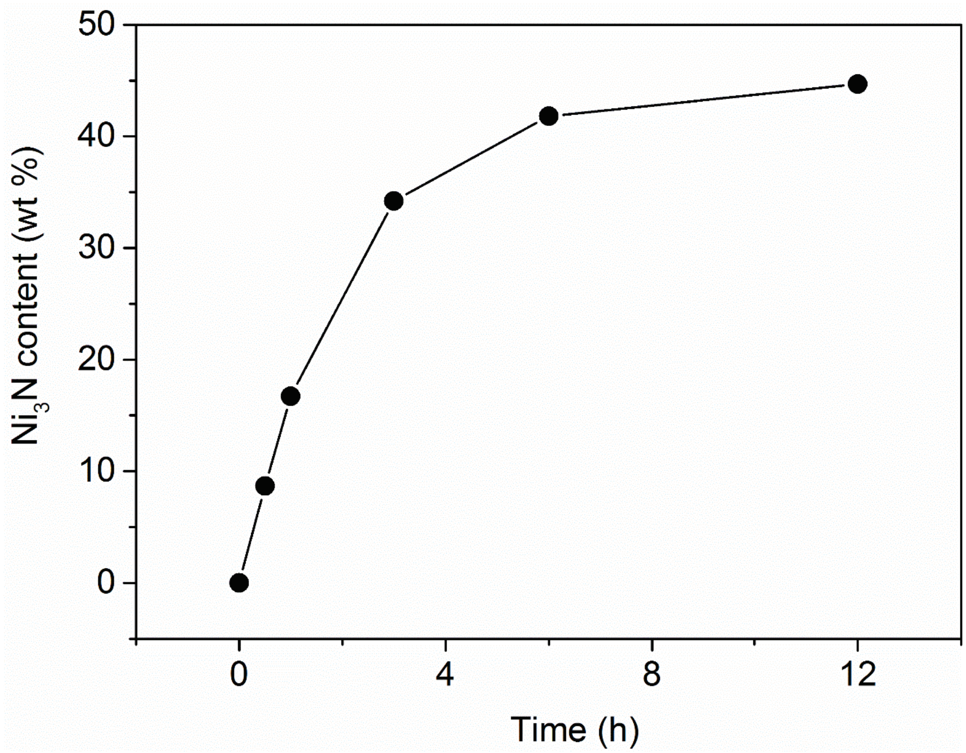

Supplementary Fig. 3. The weight percent of Ni_3N in Ni_3N/Ni/NF subjected to NH_3 treatment at 300 °C for different times.

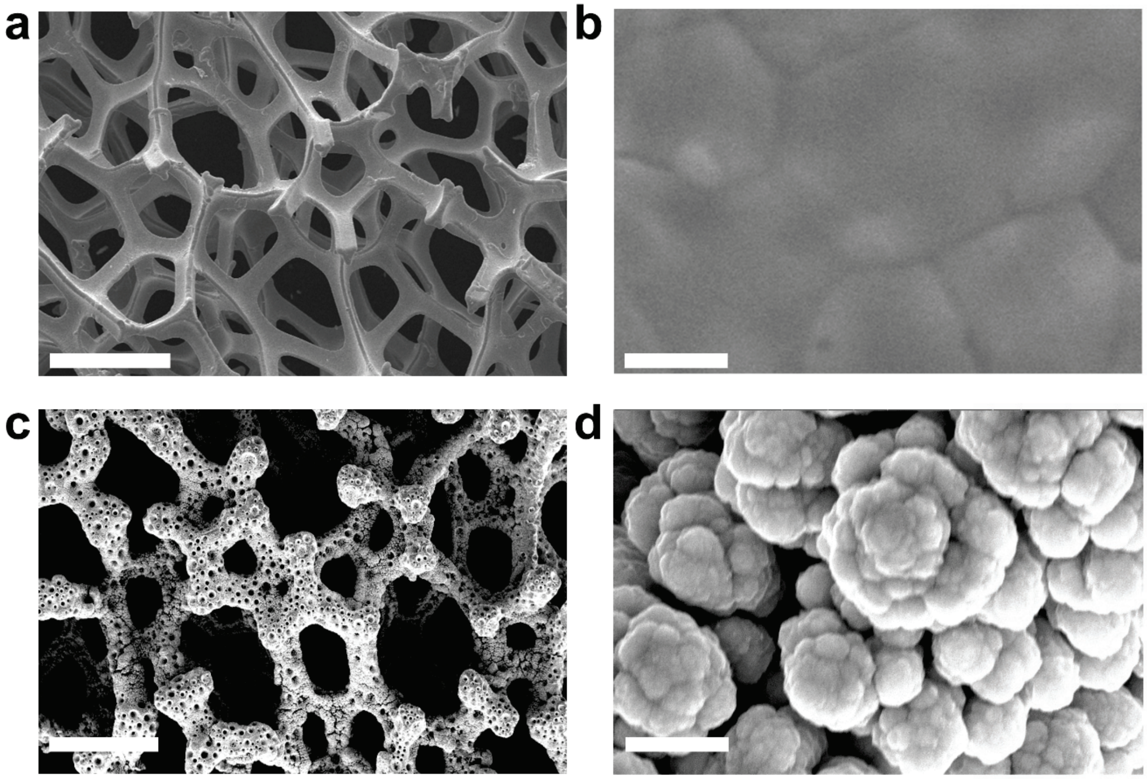

Supplementary Fig. 4. SEM characterization of NF and Ni/NF. a-b SEM images of bare NF and **c**-**d** electrodeposited Ni/NF. Scale bars, 500 μm **a**, **c**; 2 μm **b**, **d**.

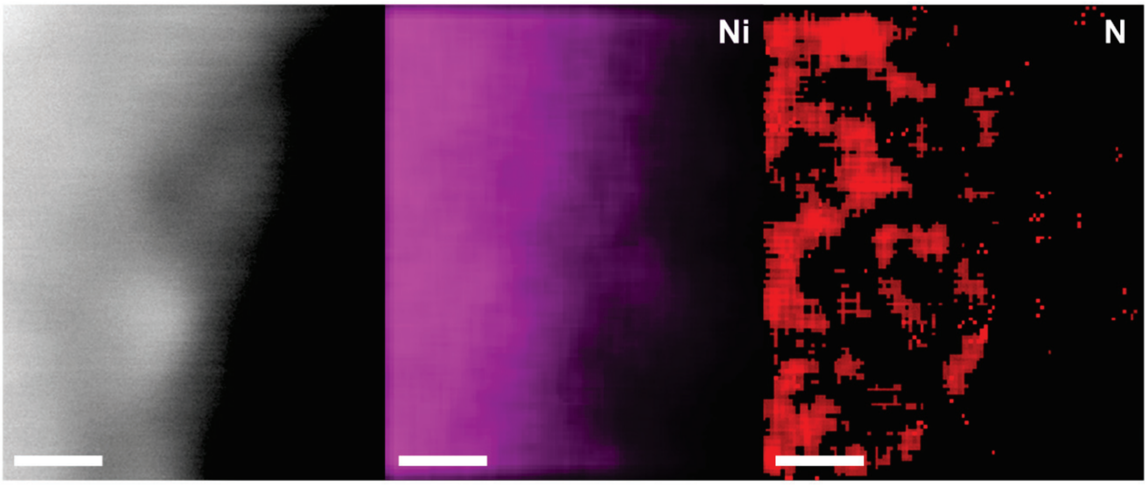

Supplementary Fig. 5. TEM and EDX mapping characterization. High-angle annular dark-field scanning transmission electron microscopy (HAADF-STEM) image and corresponding elemental maps of Ni and N in the Ni_3N/Ni interfacial electrocatalyst. Scale bar, 2.5 nm.

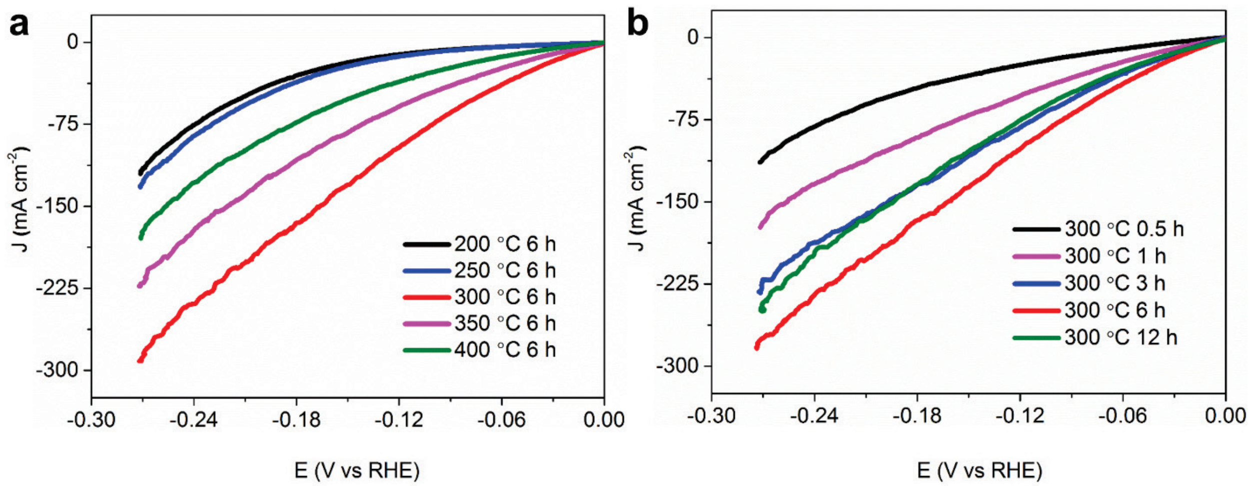

Supplementary Fig. 6. HER performance of Ni_3N/Ni/NF synthesized at different temperatures and times under neutral conditions. Linear sweep voltammetry (LSV) curves of Ni_3N/Ni/NF synthesized **a** at different nitridation temperature for 6 h and **b** at 300 °C for different duration. The LSV curves were collected in H_2-saturated 1.0 M potassium phosphate (KPi) buffer at a scan rate of 5 mV s^{-1}.

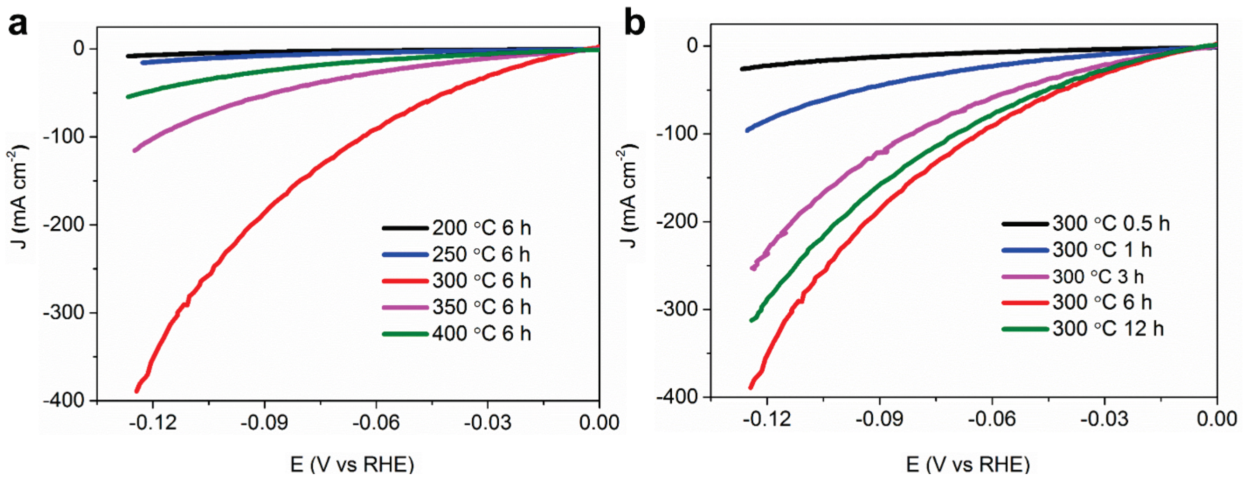

Supplementary Fig. 7. HER performance of Ni_3N/Ni/NF synthesized at different temperatures and times under alkaline conditions. Linear sweep voltammetry (LSV) curves of Ni_3N/Ni/NF synthesized **a** at different nitridation temperature for 6 h and **b** at 300 °C for different duration. The LSV curves were collected in H_2-saturated 1.0 M KOH at a scan rate of 5 mV s^{-1}.

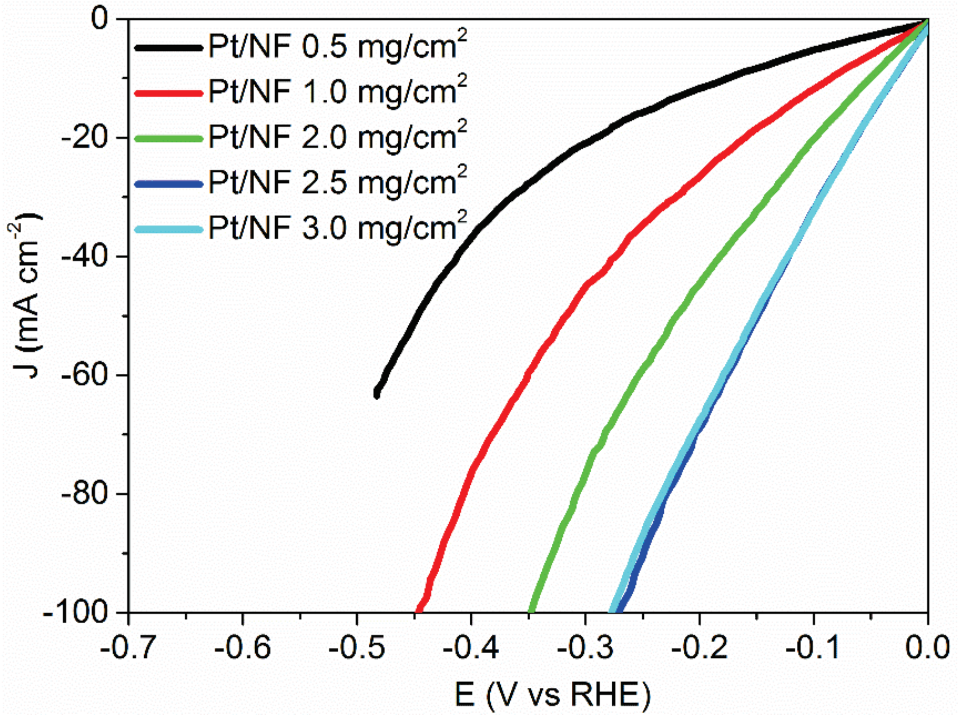

Supplementary Fig. 8. HER performance of Pt/C loaded on NF under neutral conditions. Linear sweep voltammetry curves of Pt/NF with different mass loadings of Pt/C for HER measured in H_2-saturated 1.0 M KPi.

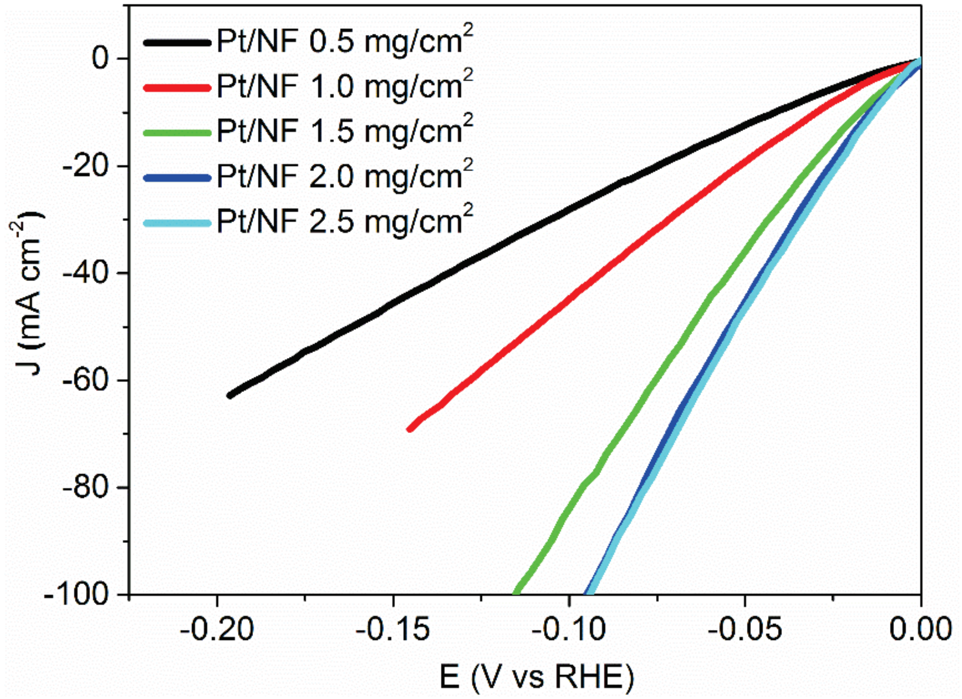

Supplementary Fig. 9. HER performance of Pt/C loaded on NF under alkaline conditions. Linear sweep voltammetry curves of Pt/NF with different mass loadings of Pt/C for HER in H_2-saturated 1.0 M KOH.

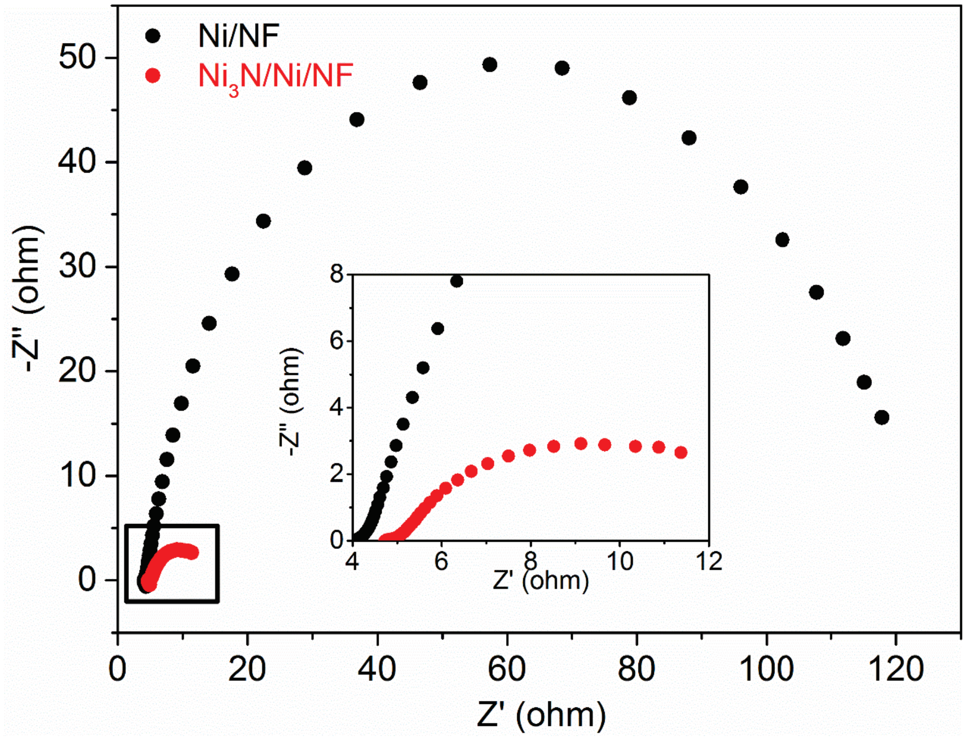

Supplementary Fig. 10. Electrochemical impedance spectroscopy (EIS) measurements under neutral conditions. Nyquist plots of Ni_3N/Ni/NF and Ni/NF measured at -0.066 V vs RHE in 1.0 M KPi buffer.

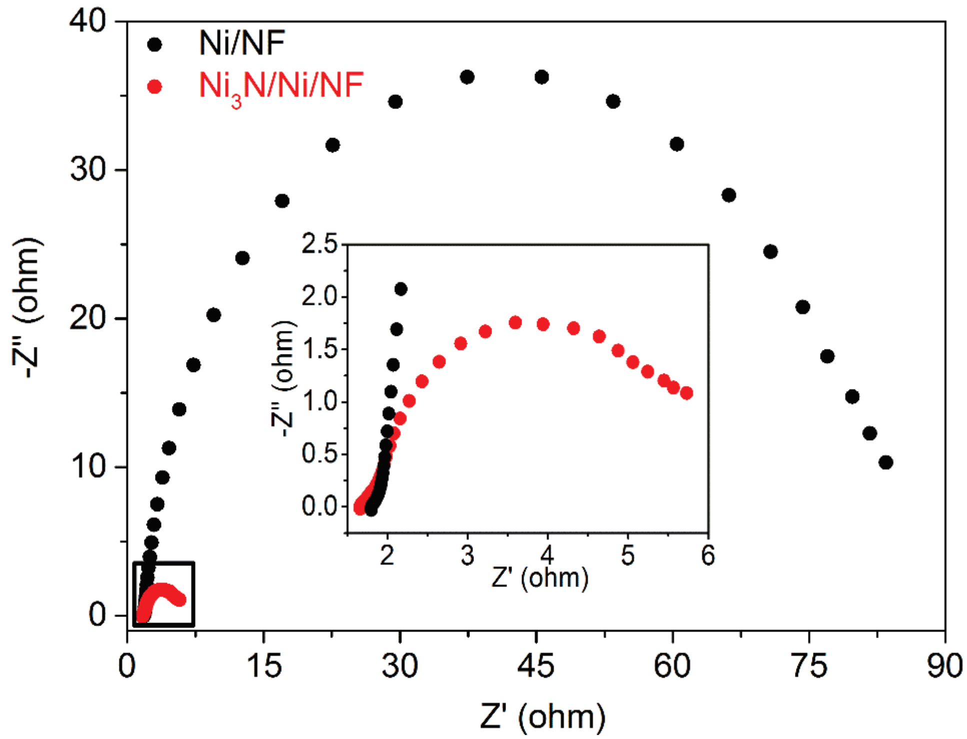

Supplementary Fig. 11. Electrochemical impedance spectroscopy (EIS) measurements under alkaline conditions. Nyquist plots of Ni_3N/Ni/NF and Ni/NF measured at -0.127 V vs RHE in 1.0 M KOH.

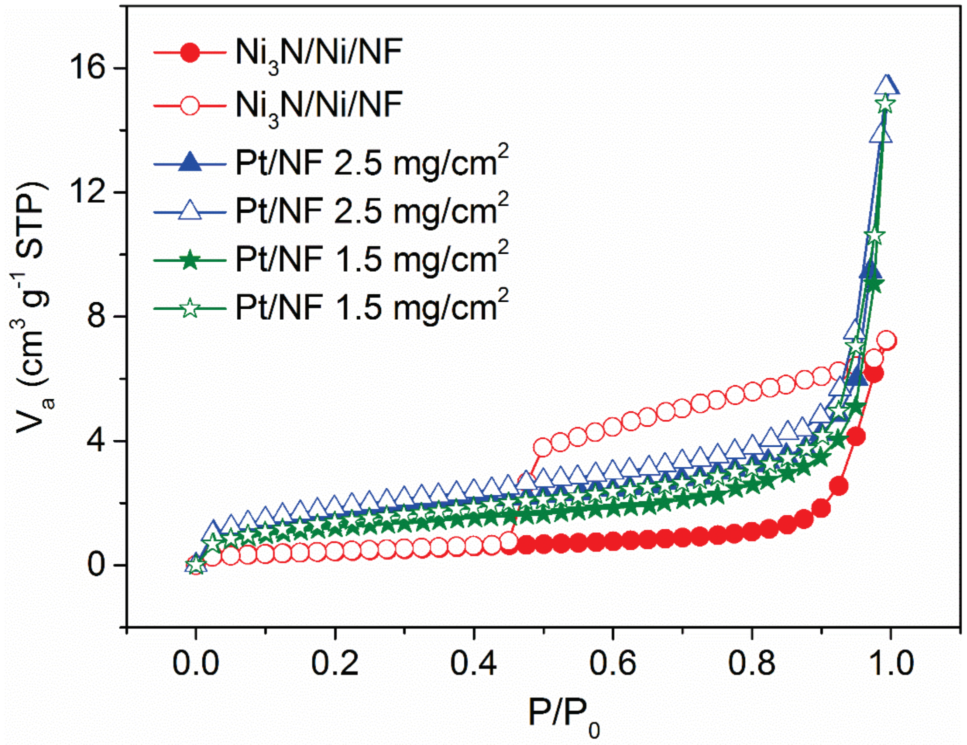

Supplementary Fig. 12. Brunauer-Emmett-Teller (BET) surface area measurements. N_2 adsorption-desorption isotherms of Ni_3N/Ni/NF, Pt/NF (1.5 mg cm^{-2}) and Pt/NF (2.5 mg cm^{-2}).

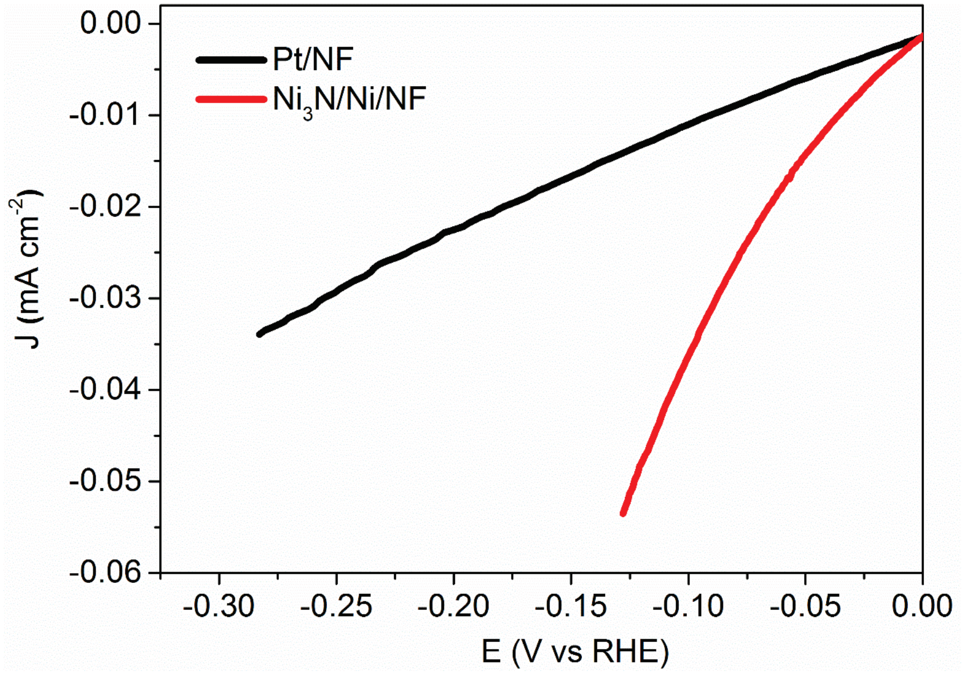

Supplementary Fig. 13. Comparison of intrinsic specific activities for HER under neutral conditions. The Brunauer-Emmett-Teller (BET) surface area-normalized linear sweep voltammetry curves of optimized Pt/NF (2.5 mg cm^{-2}) and Ni_3N/Ni/NF for HER in H_2-saturated 1.0 M KPi.

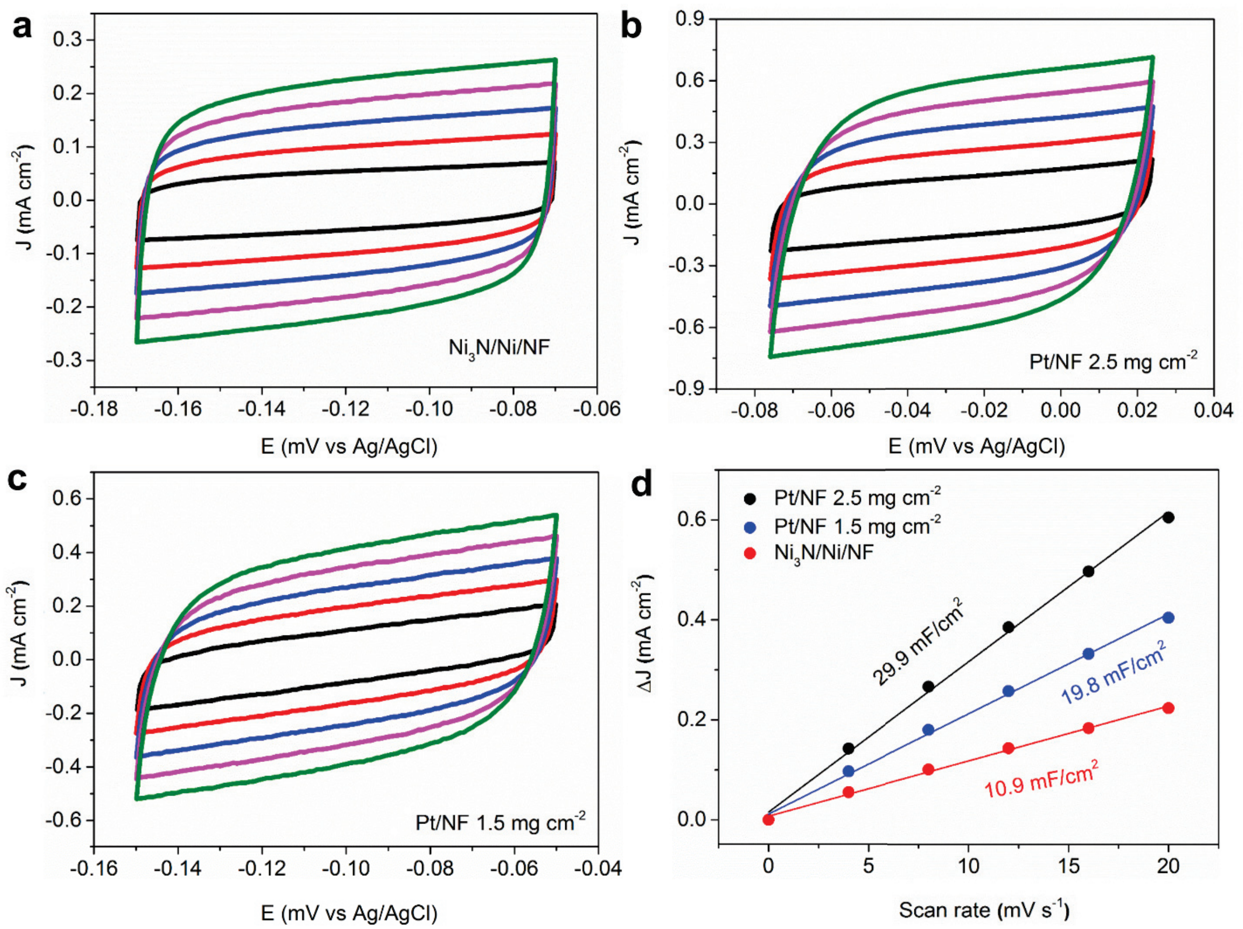

Supplementary Fig. 14. Electrochemically active surface area (ECSA) measurements. CV curves of **a** Ni_3N/Ni/NF, **b** Pt/NF (2.5 mg/cm^2), and **c** Pt/NF (1.5 mg/cm^2) collected at various scan rates ranging from 4 to 20 mV s^{-1} in CH_3CN with 0.15 M KPF_6. **d** The linear fitting of scan rate versus ΔJ (the difference between the anodic and cathodic current densities at open circuit potential).

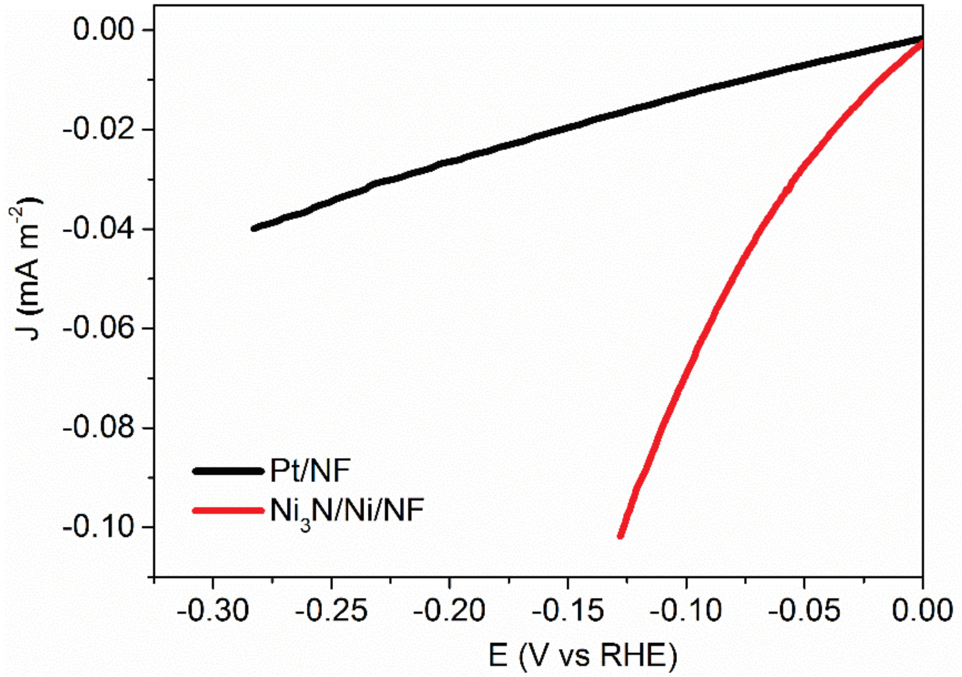

Supplementary Fig. 15. Comparison of intrinsic specific activities for HER under neutral conditions. The ECSA-normalized linear sweep voltammetry curves of optimized Pt/NF (2.5 mg/cm^2) and Ni_3N/Ni/NF for HER in H_2-saturated 1.0 M KPi.

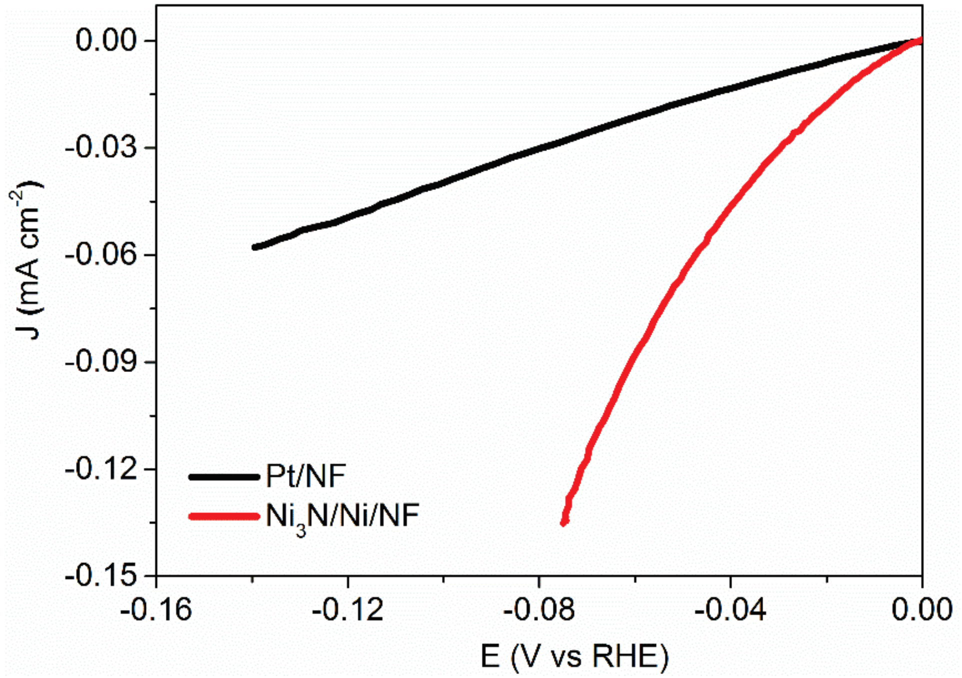

Supplementary Fig. 16. Comparison of intrinsic specific activities for HER under alkaline conditions. The ECSA-normalized linear sweep voltammetry curves of optimized Pt/NF (2.5 mg/cm^2) and $Ni_3N/Ni/NF$ for HER in H_2-saturated 1.0 M KOH.

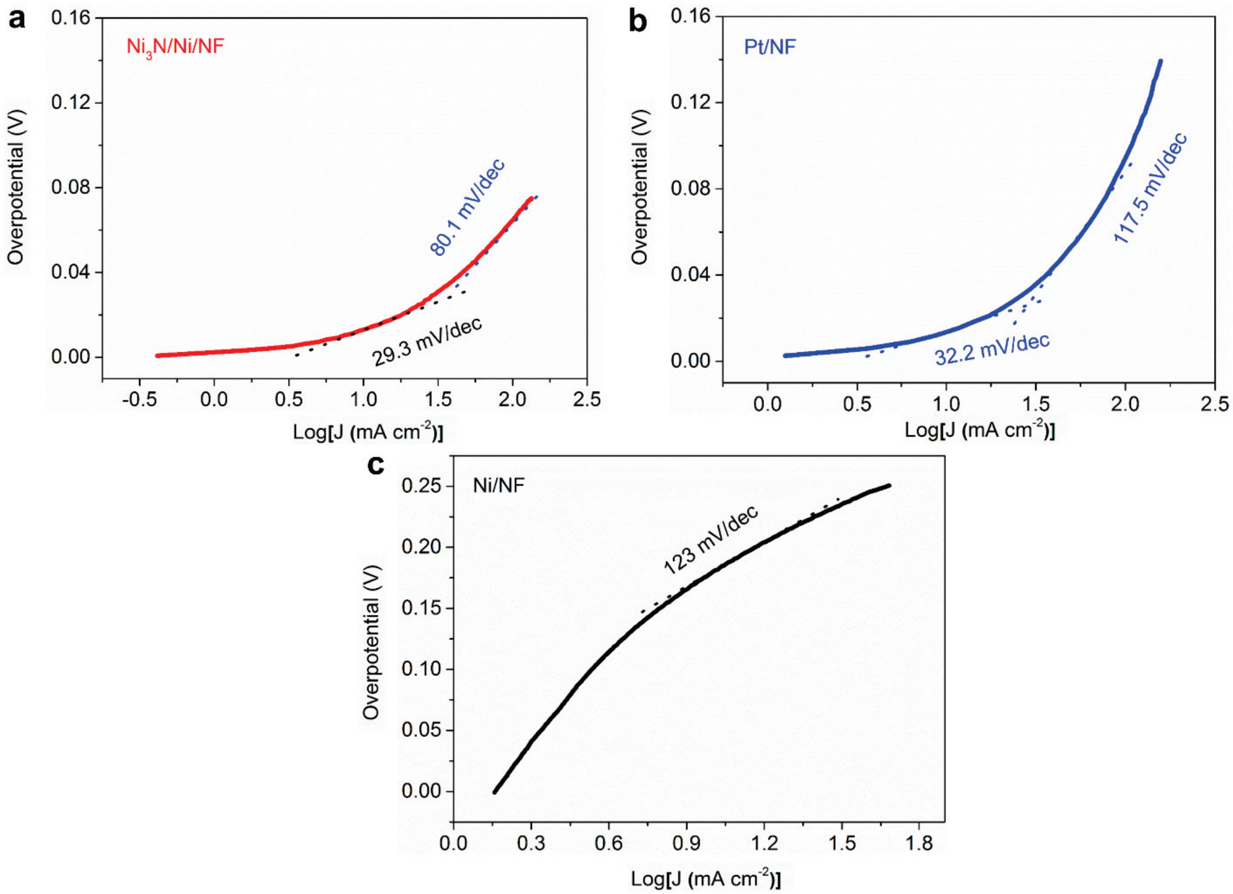

Supplementary Fig. 17. HER kinetics under alkaline conditions. Tafel plots of **a** Ni_3N/Ni/NF, **b** Pt/NF, and **c** Ni/NF with their linear fittings for HER in 1.0 M KOH.

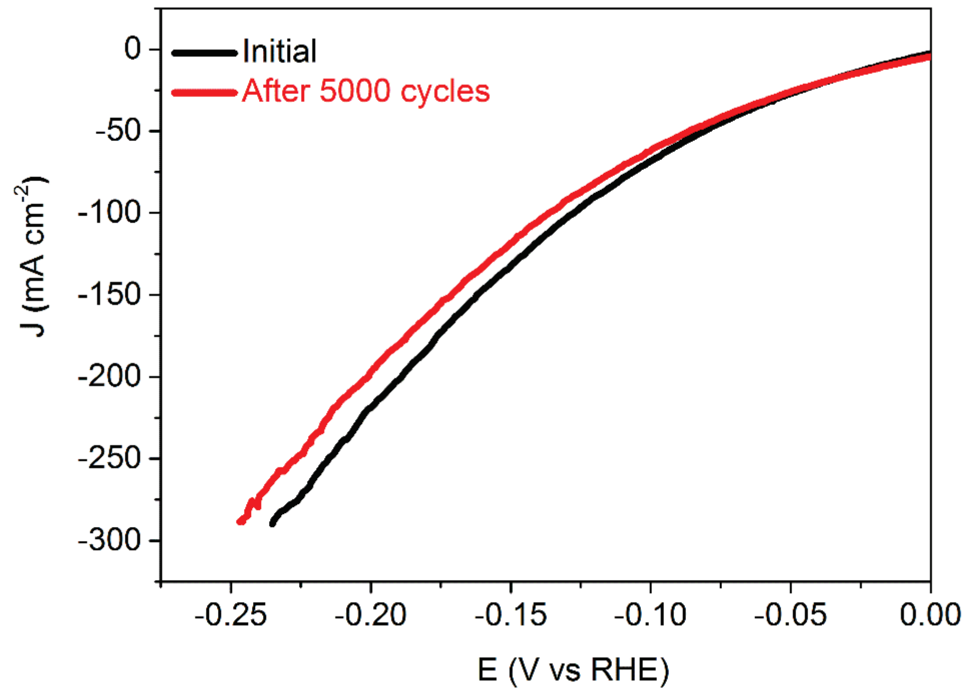

Supplementary Fig. 18. LSV curves of Ni_3N/Ni/NF before and after 5,000 CV cycles at a scan rate of 100 mV s^{-1} between 0 to -100 mV vs RHE in 1.0 M KPi buffer.

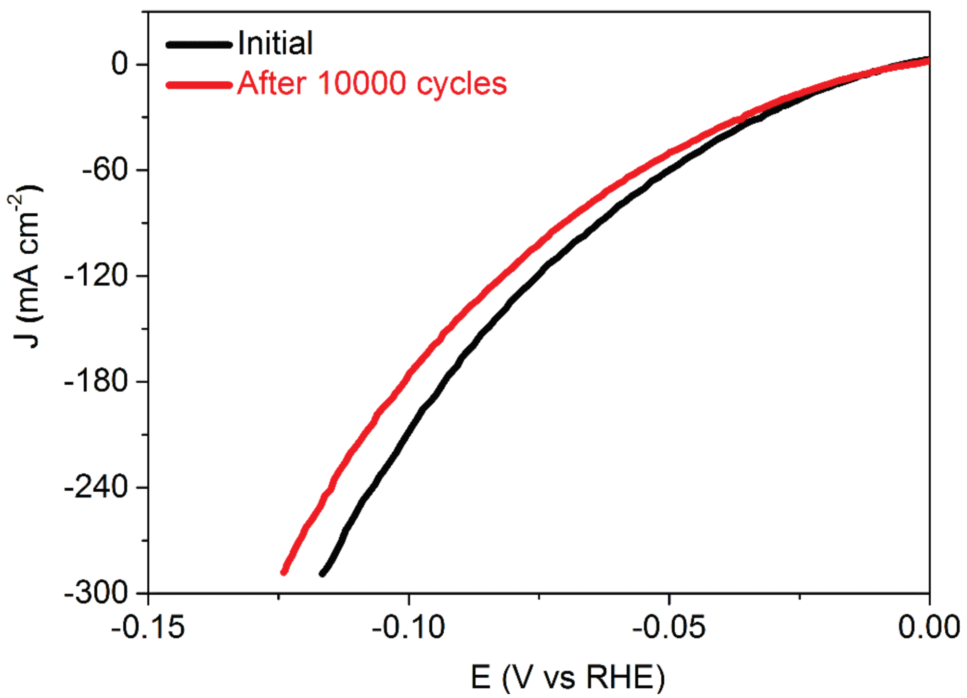

Supplementary Fig. 19. LSV curves of Ni_3N/Ni/NF before and after 10,000 cycles at a scan rate of 100 mV s^{-1} between 0 to -100 mV vs RHE in 1.0 M KOH.

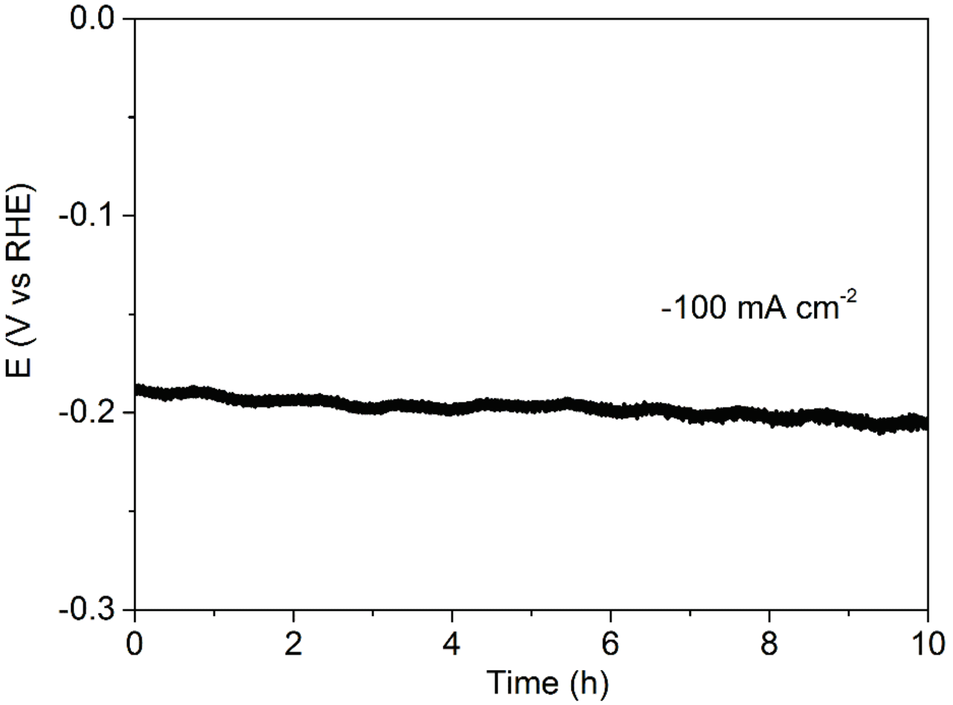

Supplementary Fig. 20. Chronopotentiometry curve of Ni_3N/Ni/NF for HER at −100 mA cm^{-2} in 1.0 M KOH.

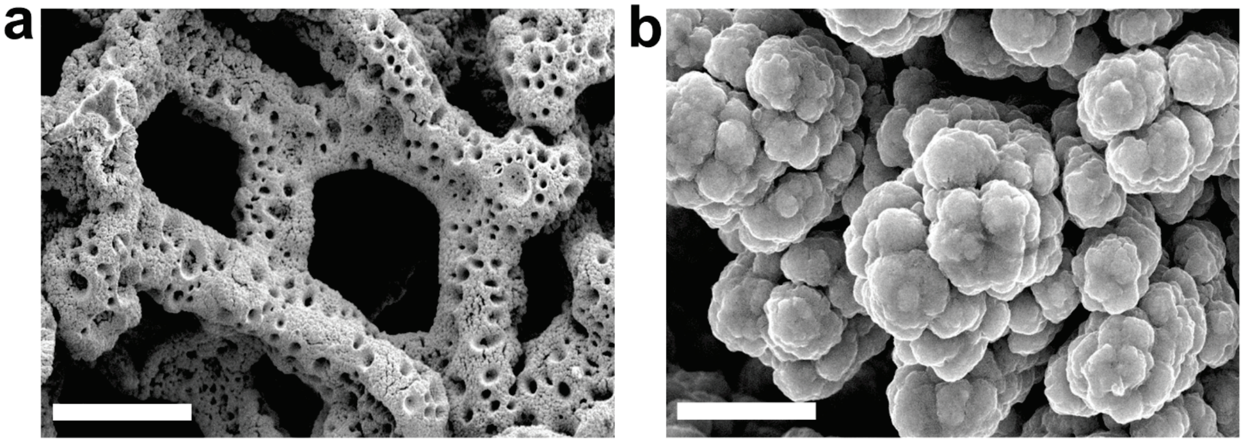

Supplementary Fig. 21. SEM characterization of post-HER Ni_3N/Ni/NF. SEM images of Ni_3N/Ni/NF after HER electrolysis at −10 mA cm^{-2} for 50 h in 1.0 M KOH. Scale bars, 200 μm **a**; 4 μm **b**.

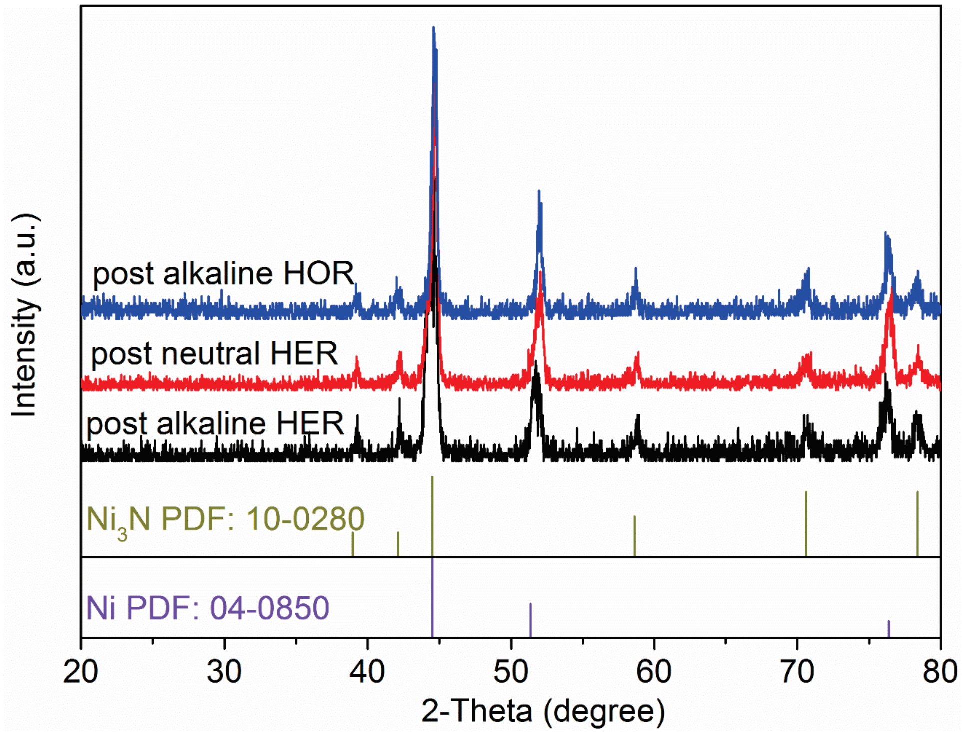

Supplementary Fig. 22. XRD patterns of post-HER/HOR Ni_3N/Ni/NF.

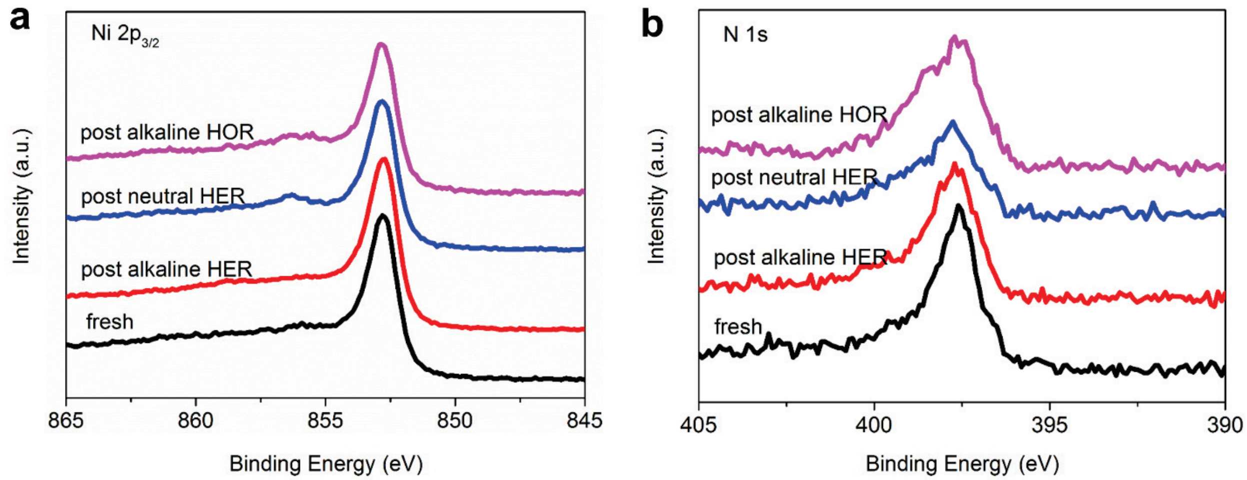

Supplementary Fig. 23. XPS spectra of **a** Ni $2p_{3/2}$ and **b** N 1s of post-HER/HOR Ni_3N/Ni/NF.

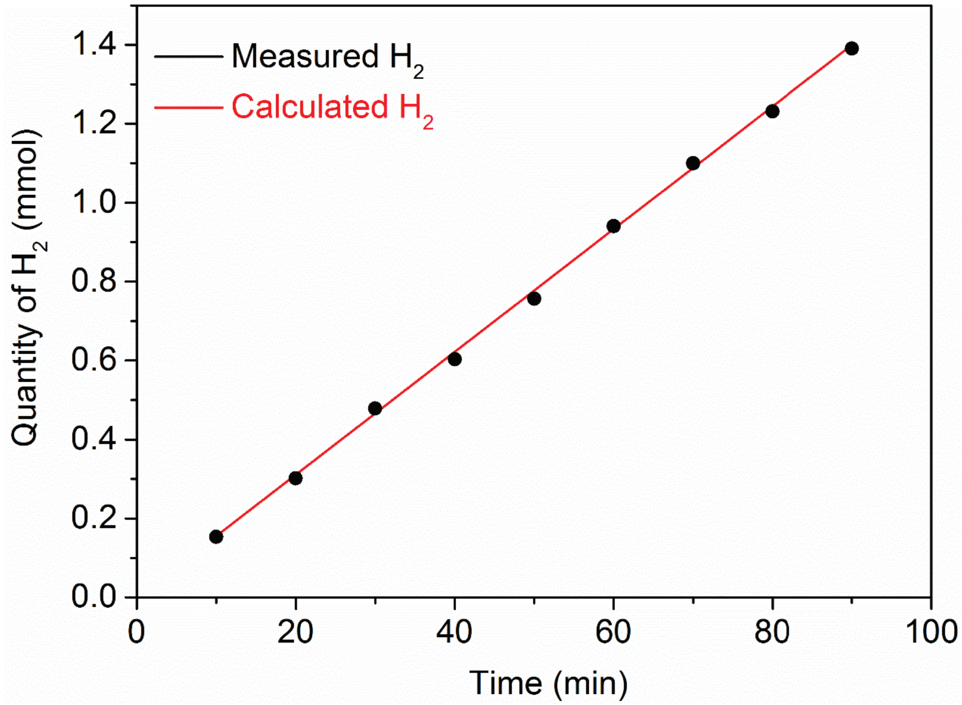

Supplementary Fig. 24. Comparison of measured and theoretically calculated H_2 amounts during the electrolysis in 1.0 M KOH at -200 mA cm^{-2}.

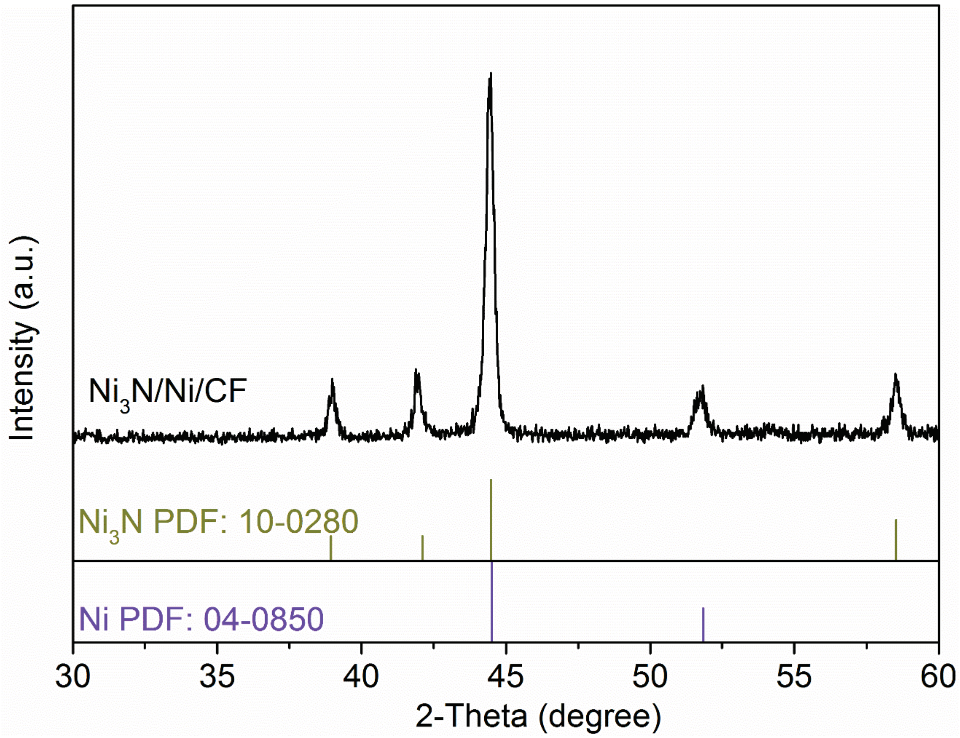

Supplementary Fig. 25. XRD pattern of Ni_3N/Ni/CF.

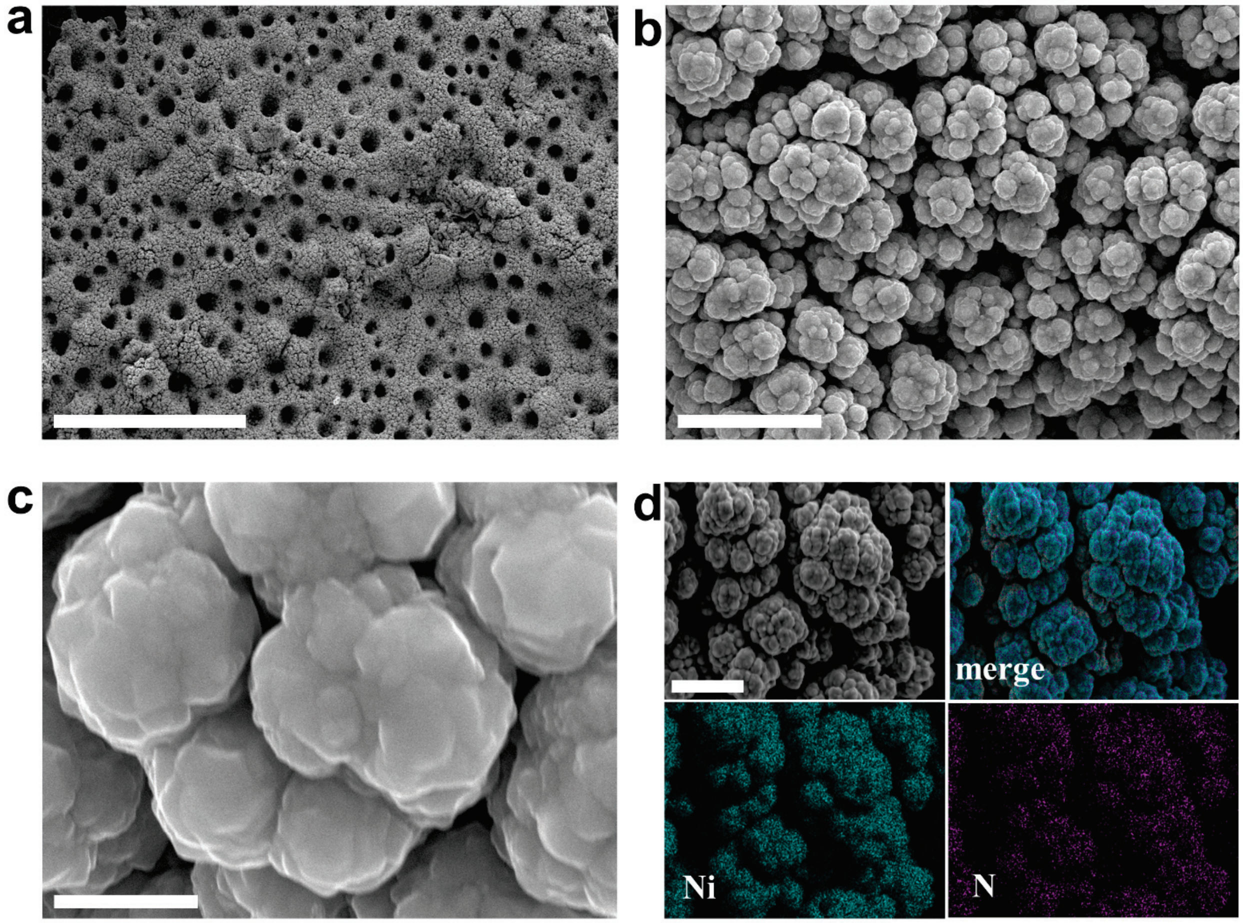

Supplementary Fig. 26. SEM and EDX mapping characterization of Ni_3N/Ni/CF. SEM images **a**-**c**, and elemental maps **d** of Ni_3N/Ni/CF. Scale bars, 500 μm **a**; 10 μm **b**; 1 μm **c**; 5 μm **d**.

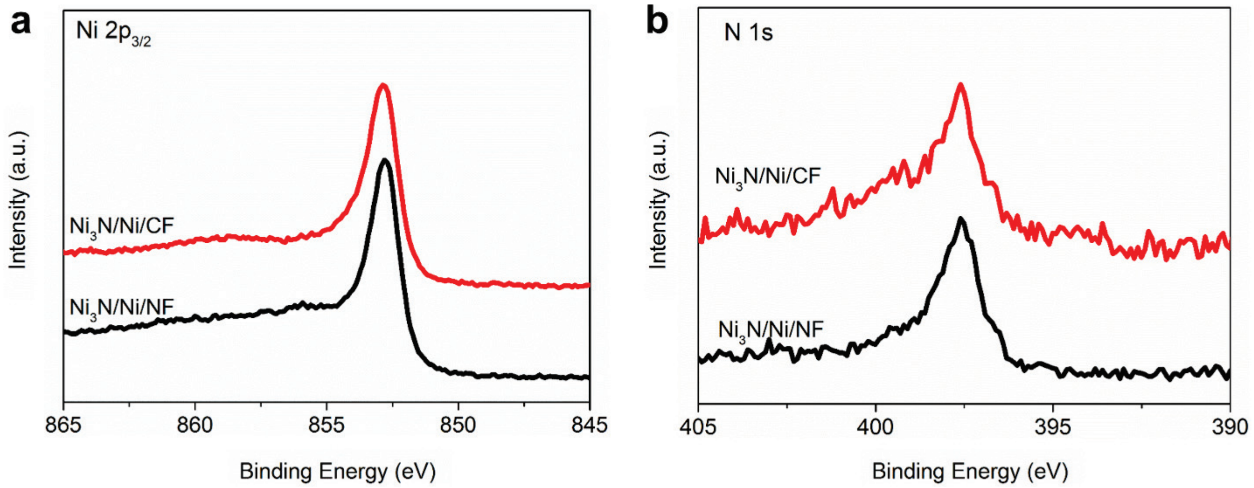

Supplementary Fig. 27. Comparison of **a** Ni $2p_{3/2}$ and **b** N 1s XPS spectra of Ni_3N/Ni/NF and Ni_3N/Ni/CF.

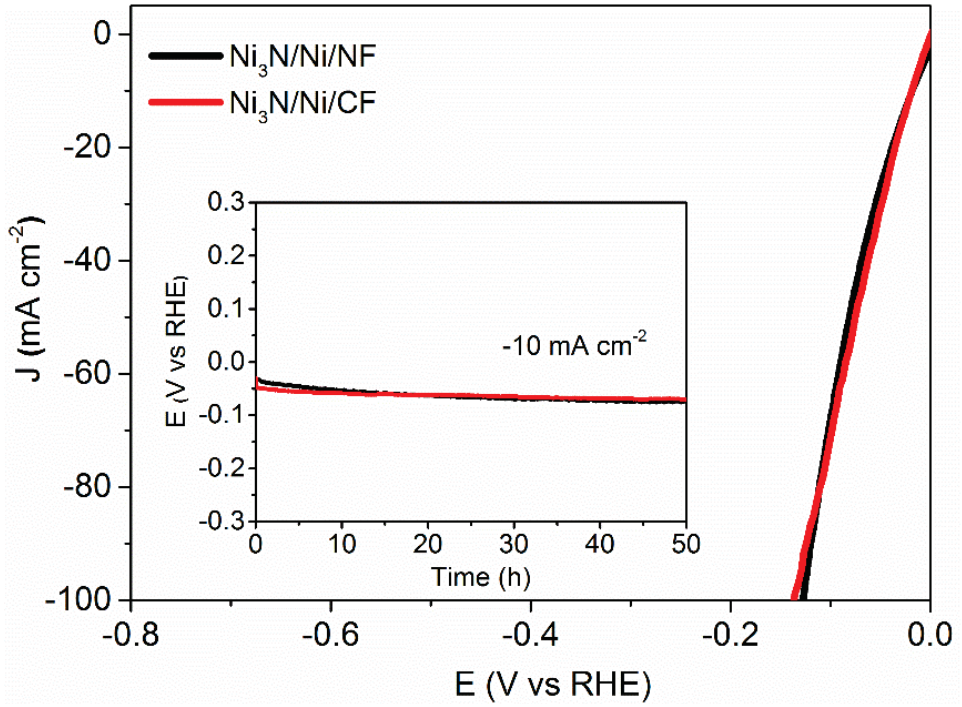

Supplementary Fig. 28. Comparison of HER performances of Ni_3N/Ni/NF and Ni_3N/Ni/CF under neutral conditions. Linear sweep voltammetry curves of Ni_3N/Ni/NF and Ni_3N/Ni/CF in 1.0 M KPi. The inset shows their chronopotentiometry curves measured at −10 mA cm^{-2}.

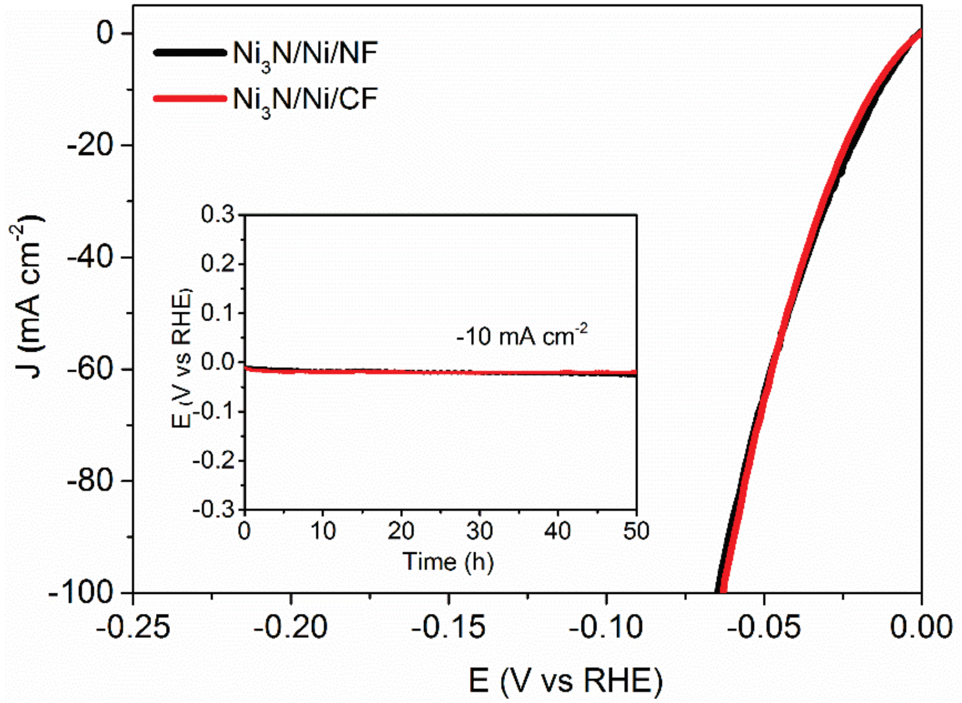

Supplementary Fig. 29. Comparison of HER performances of Ni_3N/Ni/NF and Ni_3N/Ni/CF under alkaline conditions. Linear sweep voltammetry curves of Ni_3N/Ni/NF and Ni_3N/Ni/CF in 1.0 M KOH. The inset shows their chronopotentiometry curves measured at −10 mA cm^{-2}.

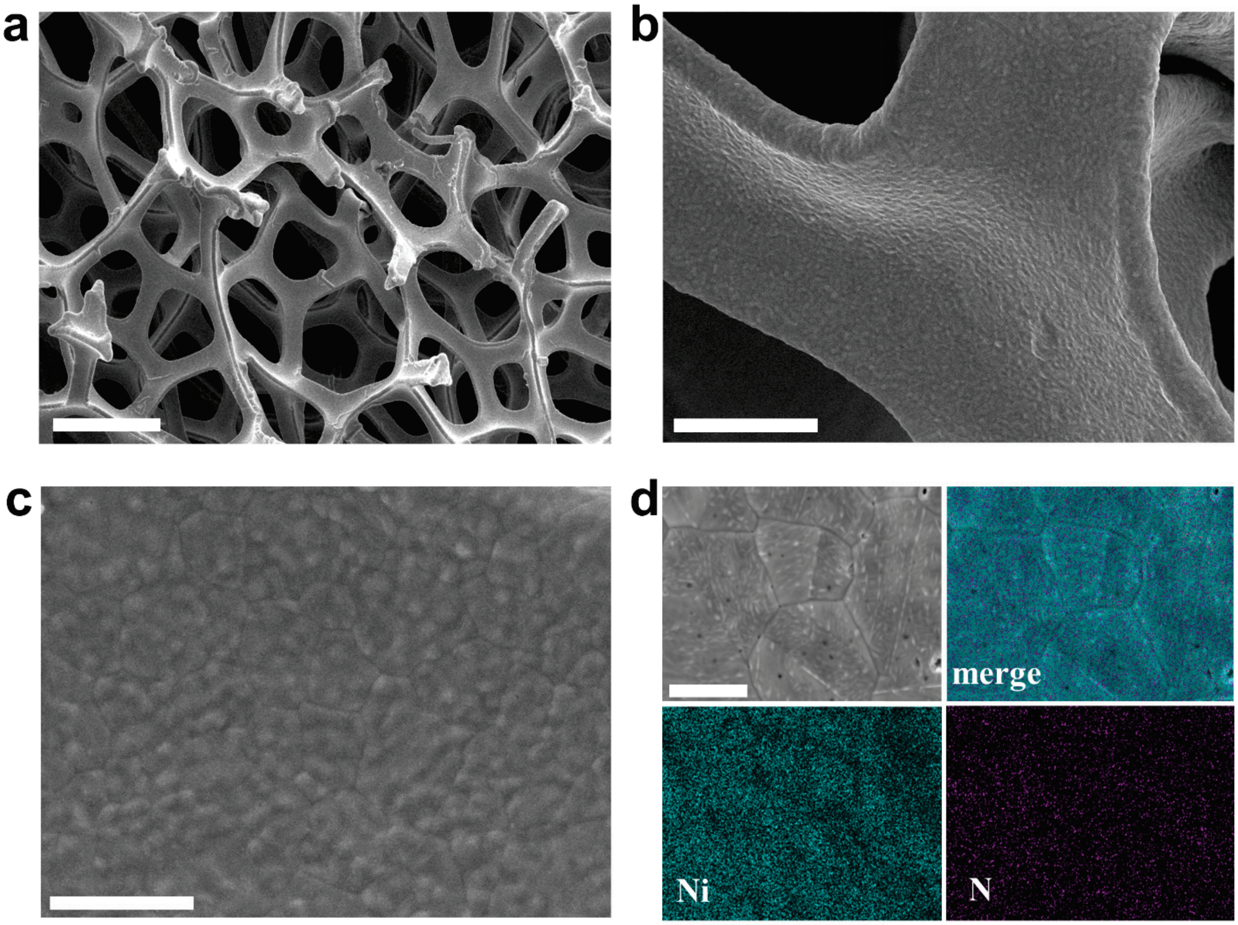

Supplementary Fig. 30. SEM and EDX mapping characterization of Ni_3N/NF. SEM images **a**-**c,** and elemental maps **d** of Ni_3N/NF. Scale bars, 500 µm **a**; 50 µm **b**; 20 µm **c**; 5 µm **d**.

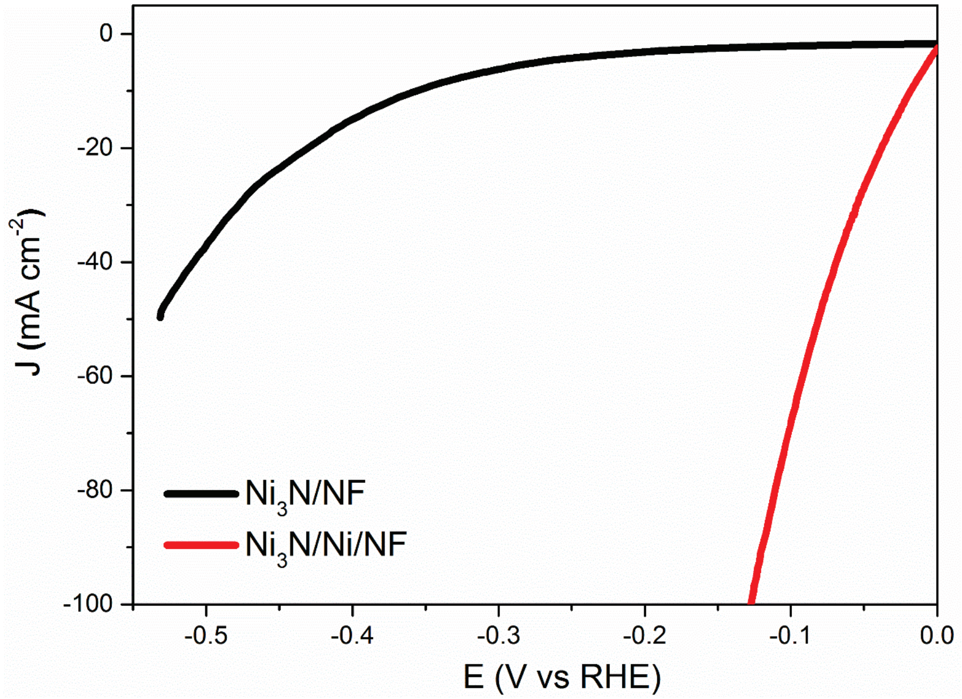

Supplementary Fig. 31. Comparison of HER performances of Ni_3N/Ni/NF and Ni_3N/NF under neutral conditions. Linear sweep voltammetry (LSV) curves of Ni_3N/Ni/NF and Ni_3N/NF for HER in H_2-saturated 1.0 M KPi.

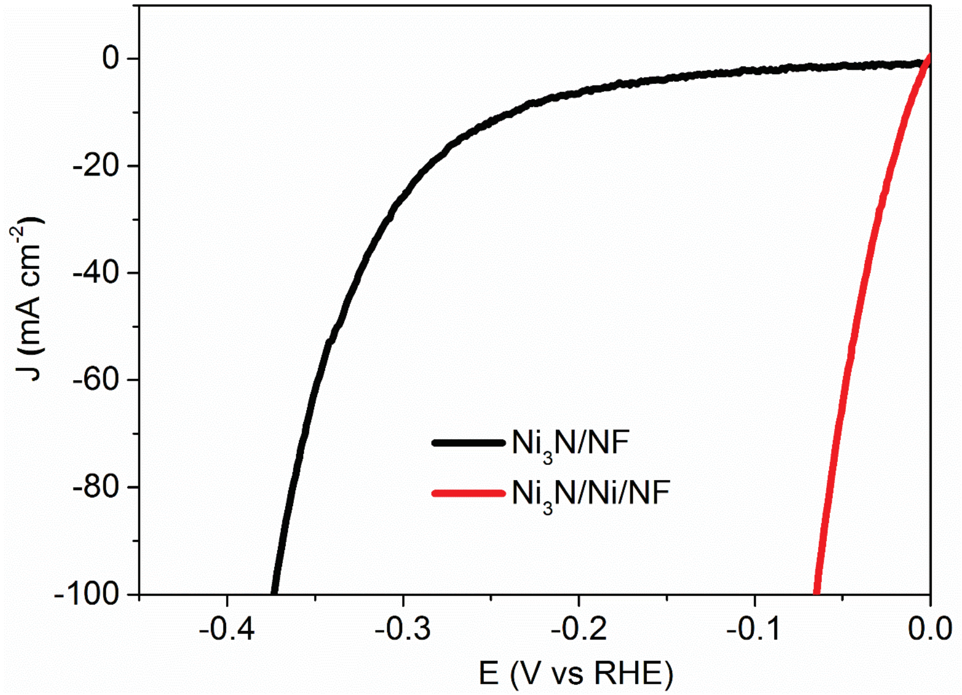

Supplementary Fig. 32. Comparison of HER performances of Ni_3N/Ni/NF and Ni_3N/NF under alkaline conditions. Linear sweep voltammetry (LSV) curves of Ni_3N/Ni/NF and Ni_3N/NF for HER in H_2-saturated 1.0 M KOH.

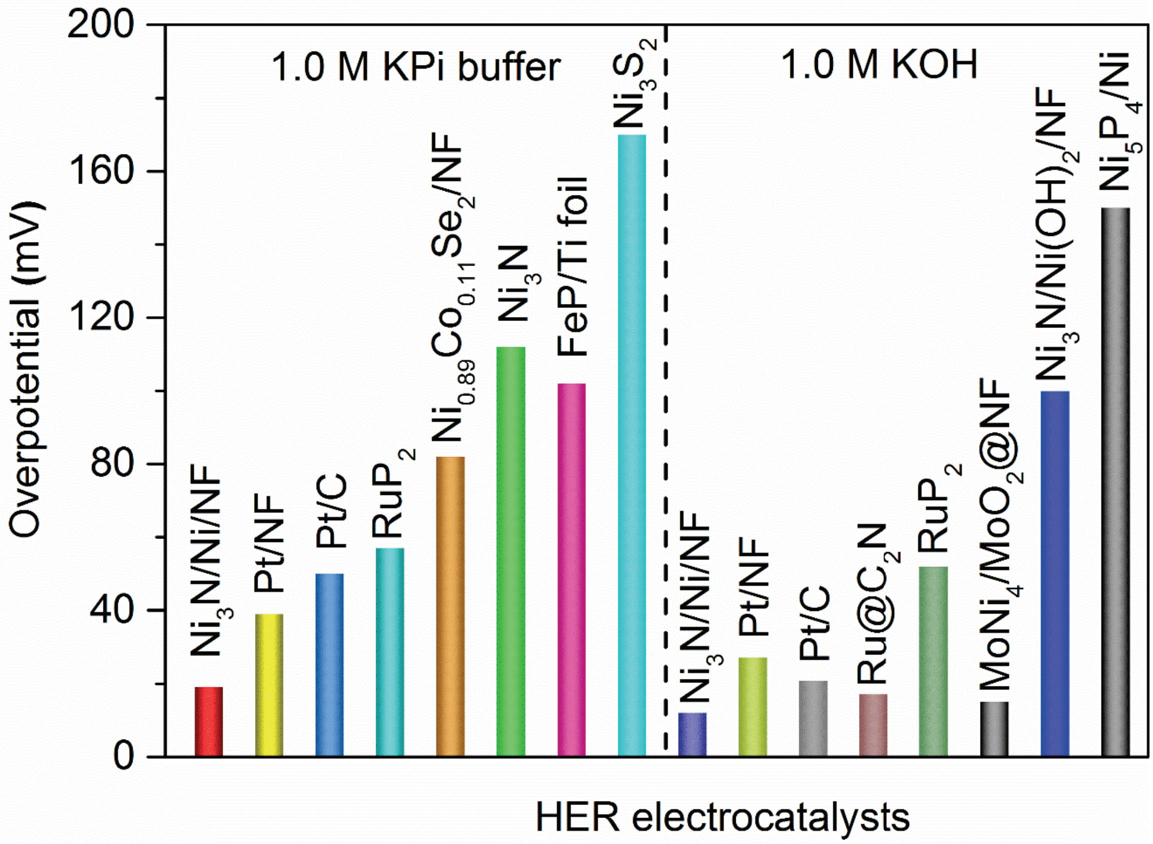

Supplementary Fig. 33. Comparison of overpotential requirement to deliver −10 mA cm^{-2} for HER electrocatalysts in various electrolytes. Data are summarized in **Supplementary Table 3**.

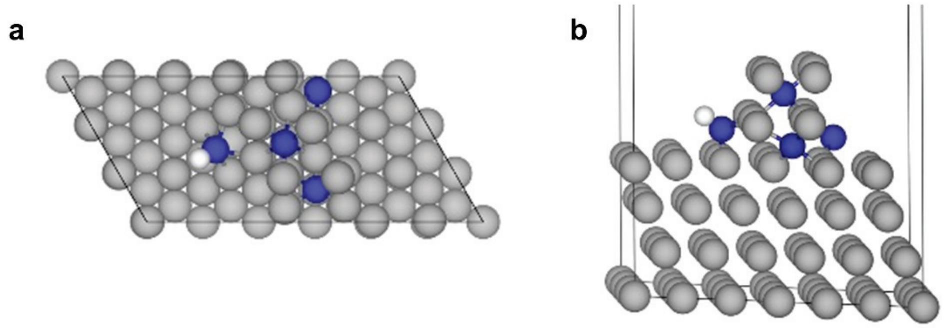

Supplementary Fig. 34. H adsorption structures. **a** Top view and **b** side view of Ni_3N/Ni_N. Color code: Ni: grey; N: blue; H: white.

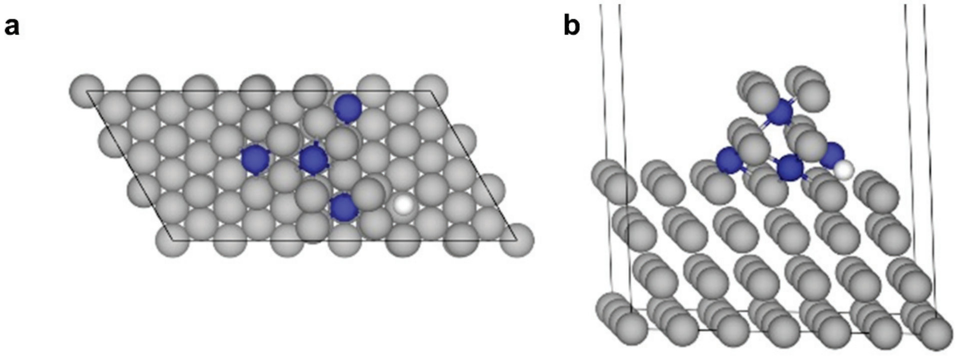

Supplementary Fig. 35. H adsorption structures. **a** Top view and **b** side view of Ni_3N/Ni_hollow. Color code: Ni: grey; N: blue; H: white.

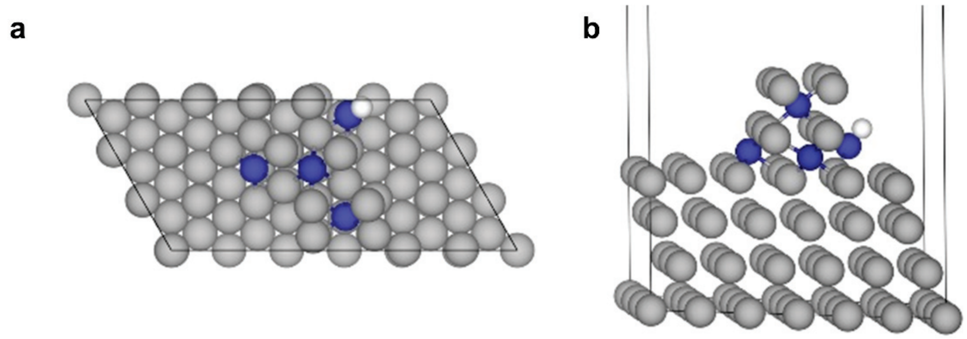

Supplementary Fig. 36. H adsorption structures. a Top view and **b** side view of Ni_3N/Ni_N_2. Color code: Ni: grey; N: blue; H: white.

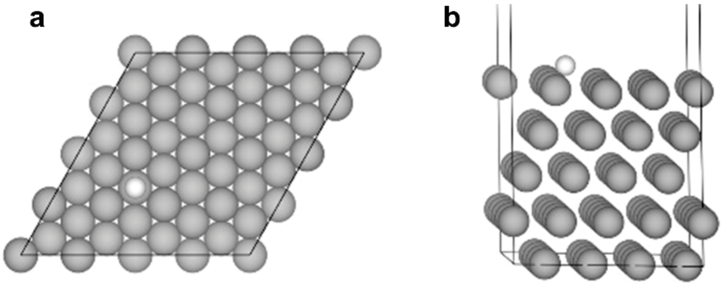

Supplementary Fig. 37. H adsorption structures. a Top view and **b** side view of Ni. H is on top of three-fold hollow site of Ni. There is a Ni beneath H in the *third* Ni layer. Color code: Ni: grey; H: white.

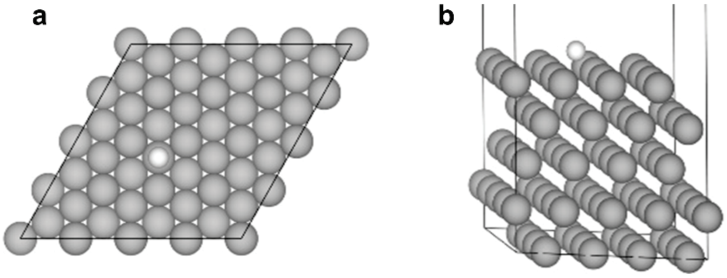

Supplementary Fig. 38. H adsorption structures. a Top view and **b** side view of Ni_2. H is on top of three-fold hollow site of Ni. There is a Ni beneath H in the *second* Ni layer. Color code: Ni: grey; H: white.

a b

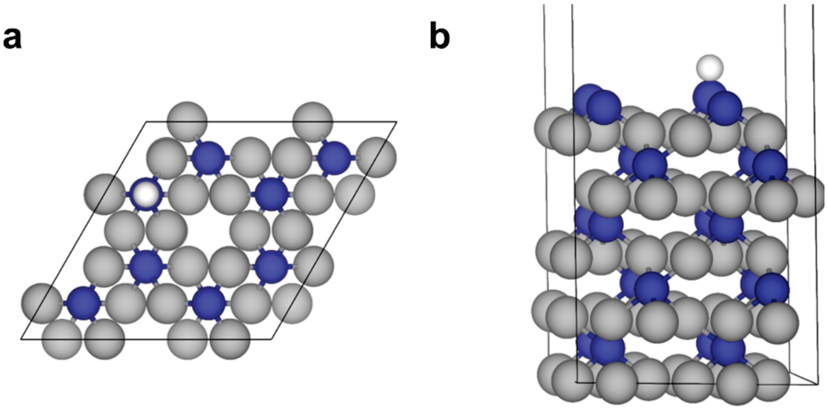

Supplementary Fig. 39. H adsorption structures. a Top view and **b** side view of Ni_3N_N. Color code: Ni: grey; N: blue; H: white.

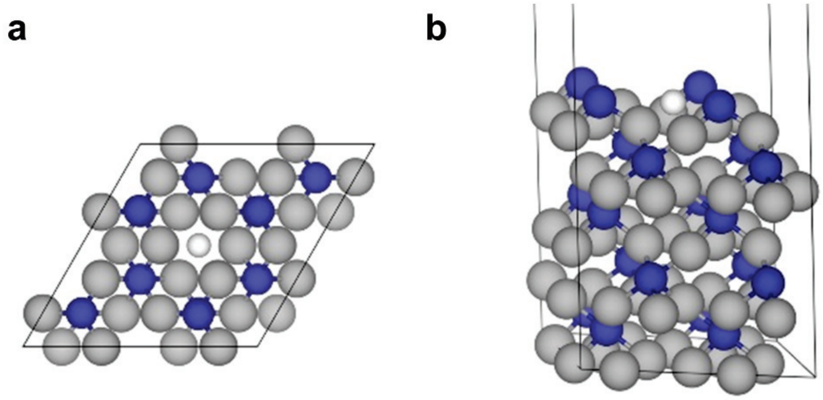

Supplementary Fig. 40. H adsorption structures. a Top view and **b** side view of Ni_3N_hollow. Color code: Ni: grey; N: blue; H: white.

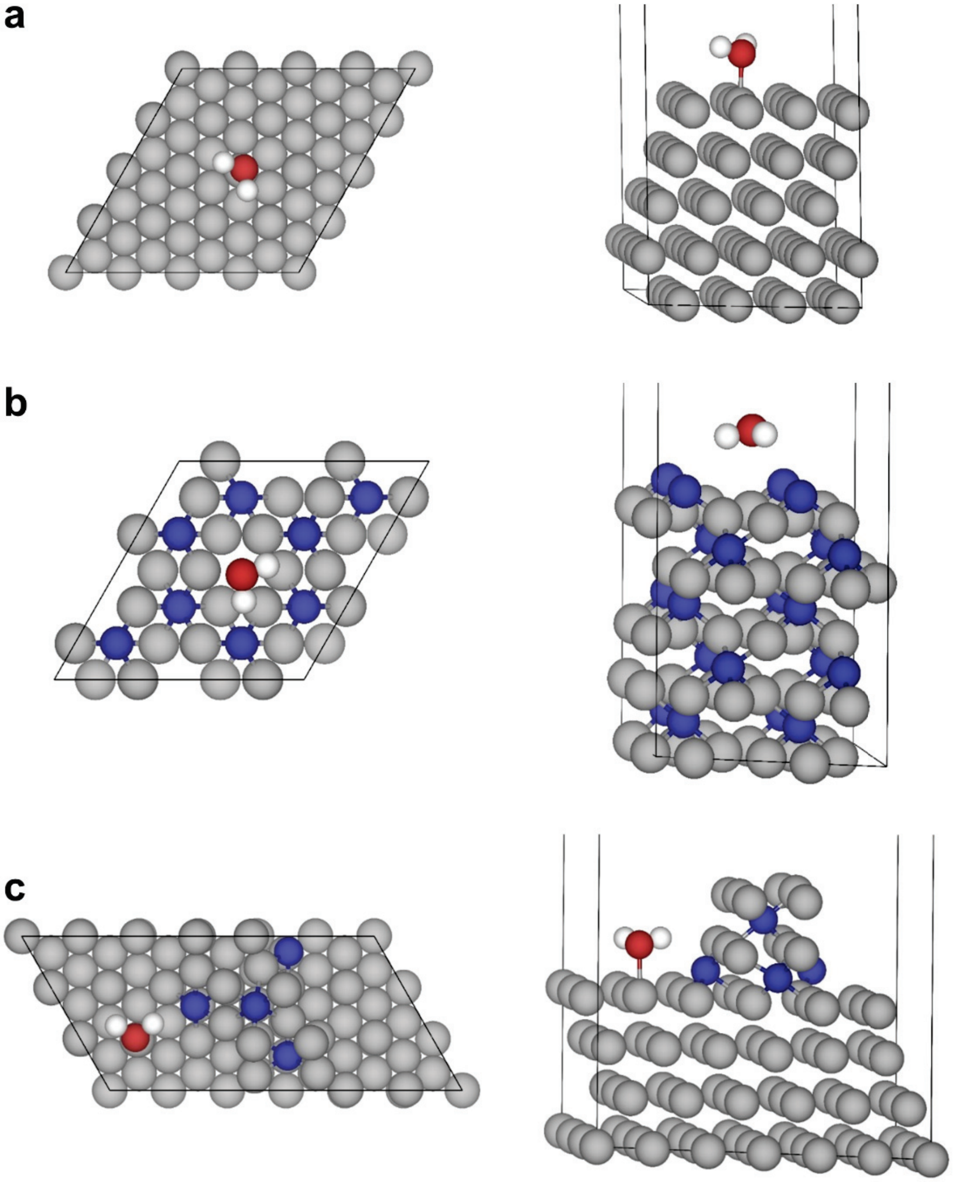

Supplementary Fig. 41. H_2O adsorption structures. Top view and side view for water adsorption on **a** Ni, **b** Ni_3N, and **c** Ni_3N/Ni interface. Color code: Ni: grey; N: blue; H: white; O: red.

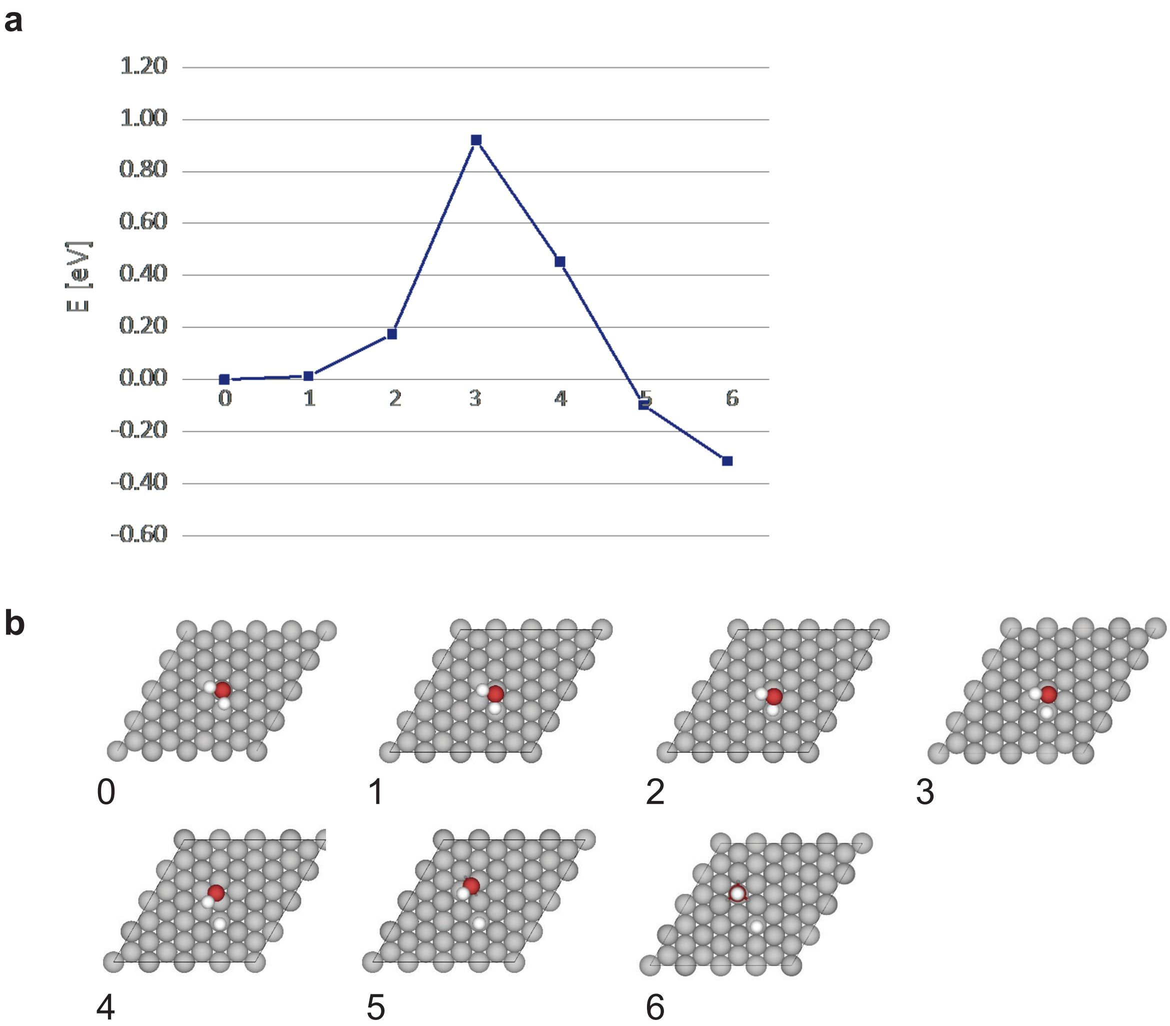

Supplementary Fig. 42. H_2O dissociation pathways. a Energy pathway for water dissociation on Ni(111). **b** Top view of intermediate structures. Ni (111), barrier = 0.92 eV. Color code: Ni: grey; H: white; O: red.

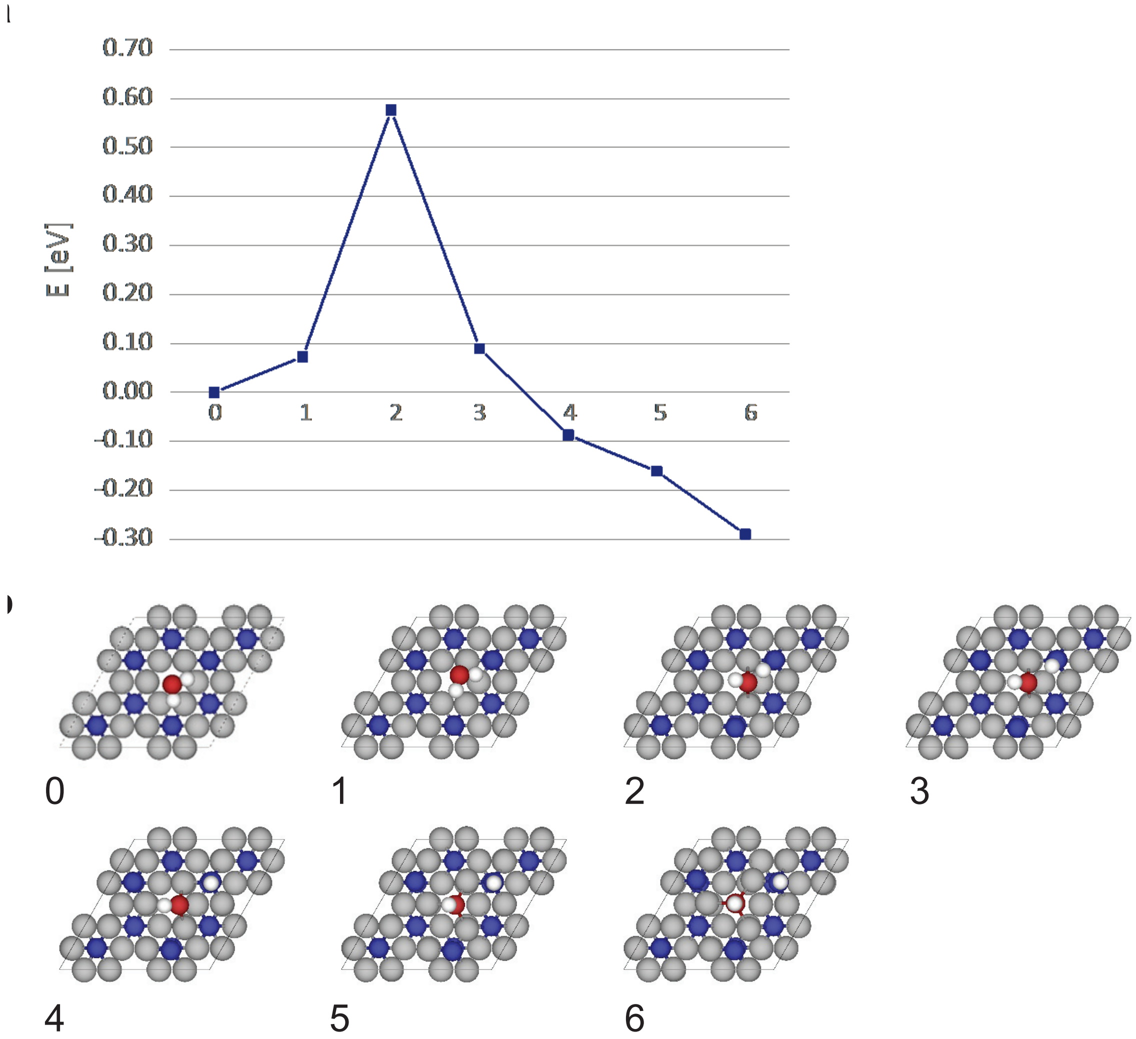

Supplementary Fig. 43. H_2O dissociation pathways. a Energy pathway for water dissociation on Ni_3N (001). **b** Top view of intermediate structures. Ni_3N (001), barrier = 0.58 eV. Color code: Ni: grey; N: blue; H: white; O: red.

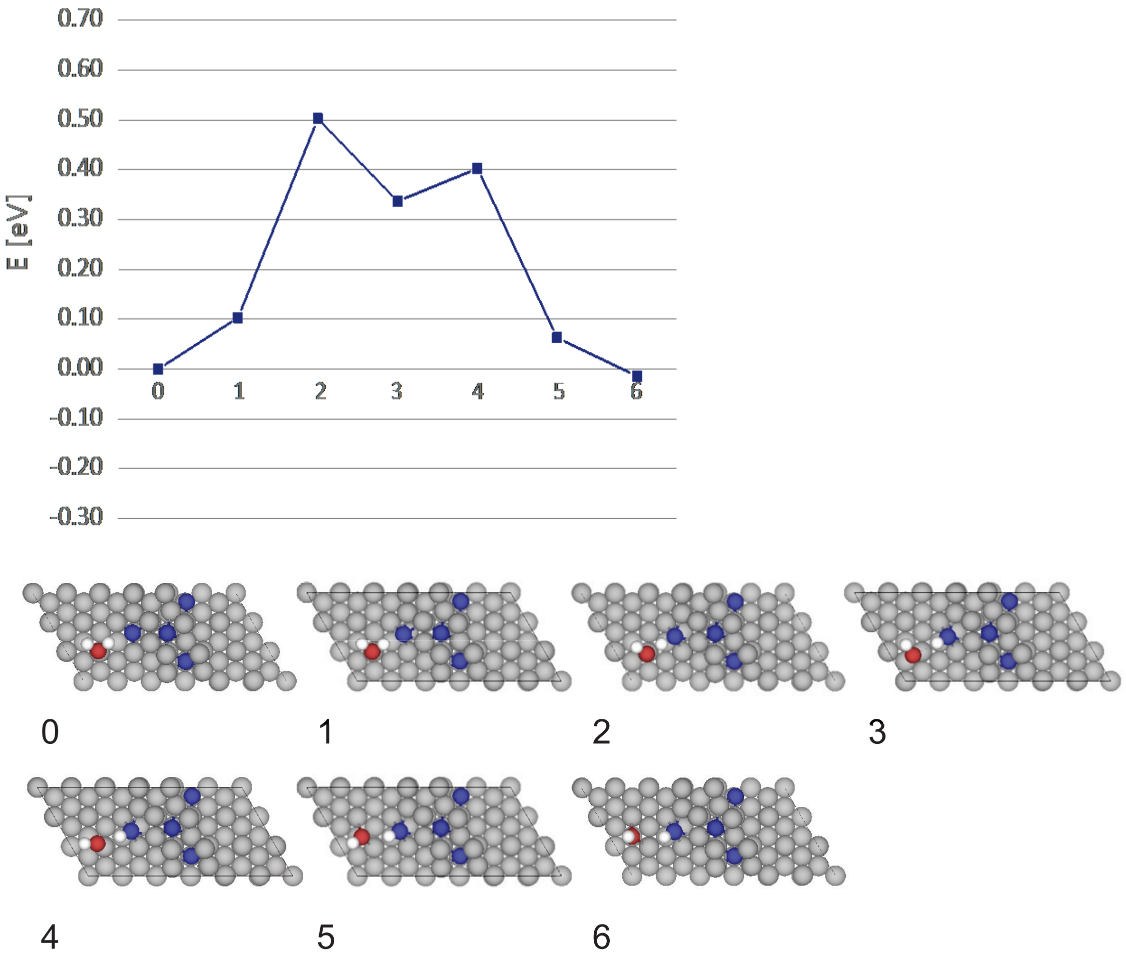

Supplementary Fig. 44. H_2O dissociation pathways. a Energy pathway for water dissociation on hybrid structure. **b** Top view of intermediate structures. Hybrid structure, barrier = 0.50 eV. Color code: Ni: grey; N: blue; H: white; O: red.

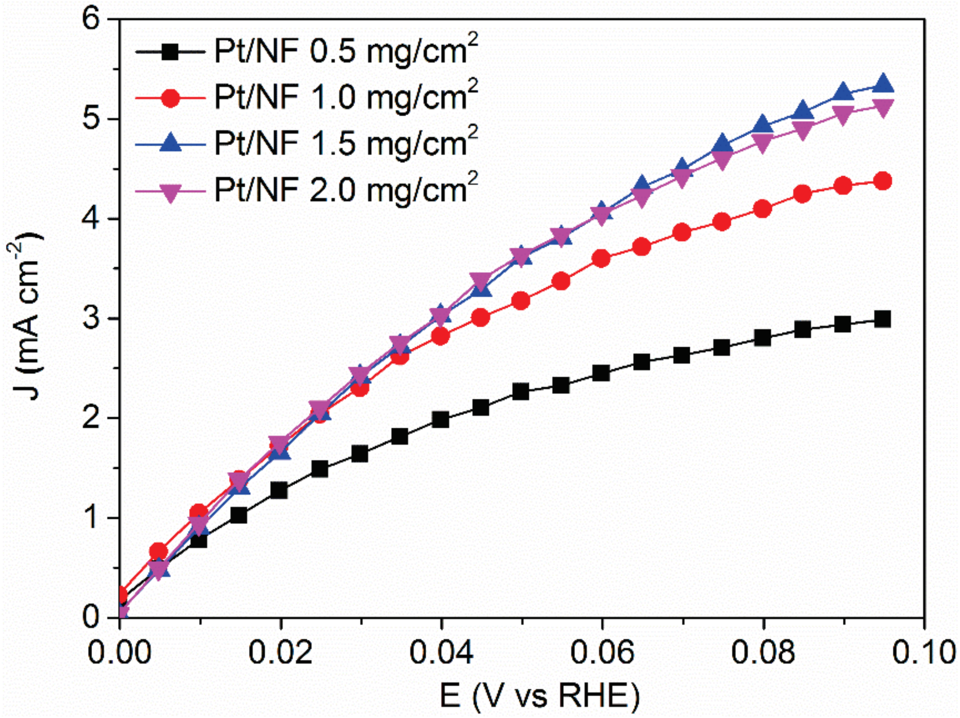

Supplementary Fig. 45. HOR performance of Pt/C loaded on NF under alkaline conditions. Steady-state polarization curves Pt/NF with different mass loadings of Pt/C for HOR in H_2-saturated 0.1 M KOH.

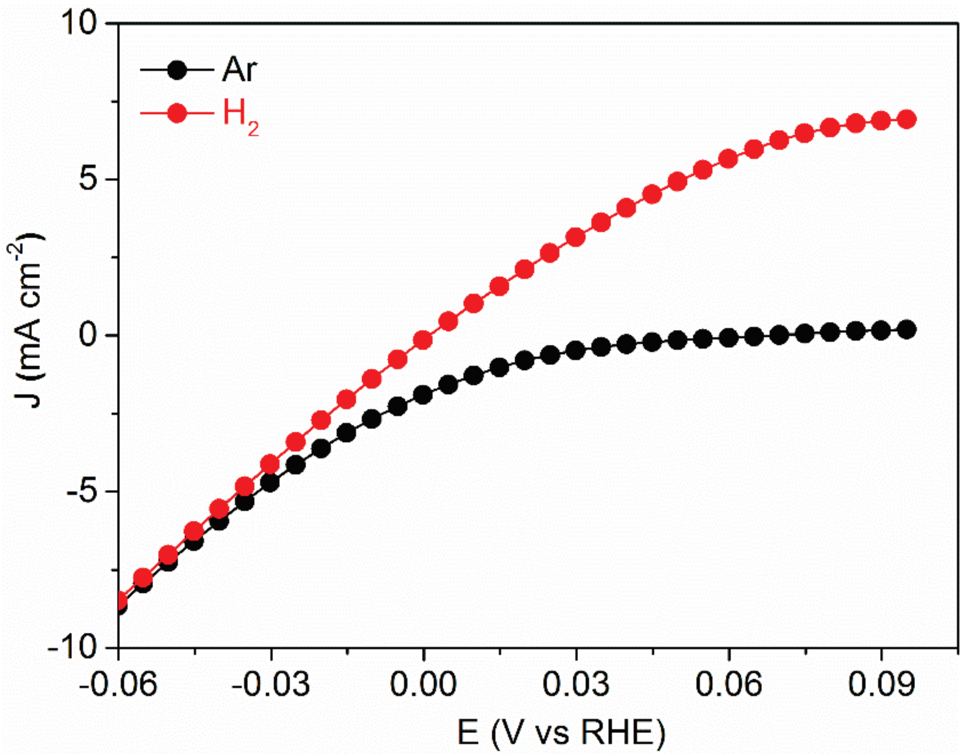

Supplementary Fig. 46. Steady-state polarization curves of Ni_3N/Ni/NF in Ar and H_2-saturated 0.1 M KOH.

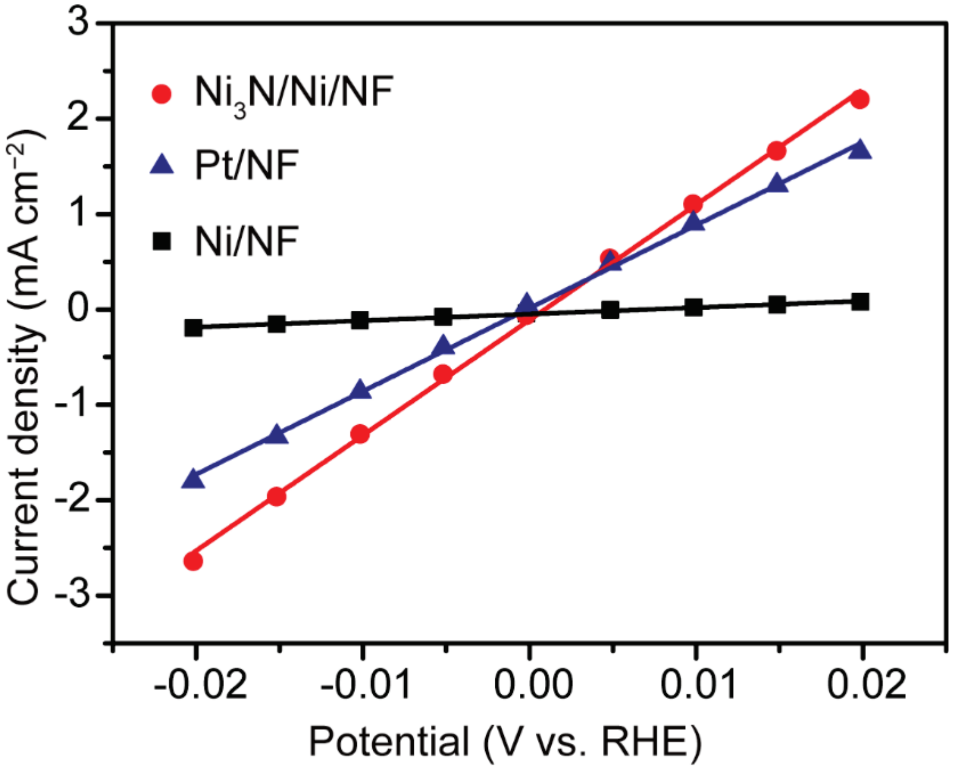

Supplementary Fig. 47. The steady-state polarization curves of Ni_3N/Ni/NF, Pt/NF, and Ni/NF in the micro-polarization region (-20 mV to 20 mV vs. RHE) in H_2-saturated 0.1 M KOH.

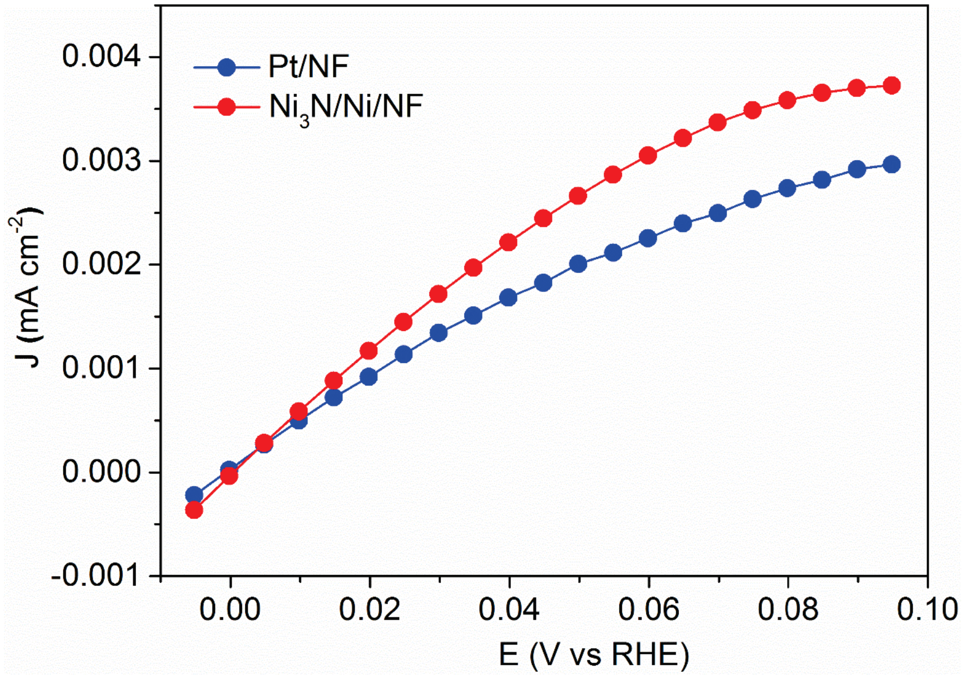

Supplementary Fig. 48. The BET surface area-normalized steady-state polarization curves of optimized Pt/NF (1.5 mg cm^{-2}) and Ni_3N/Ni/NF for HOR in H_2-saturated 0.1 M KOH.

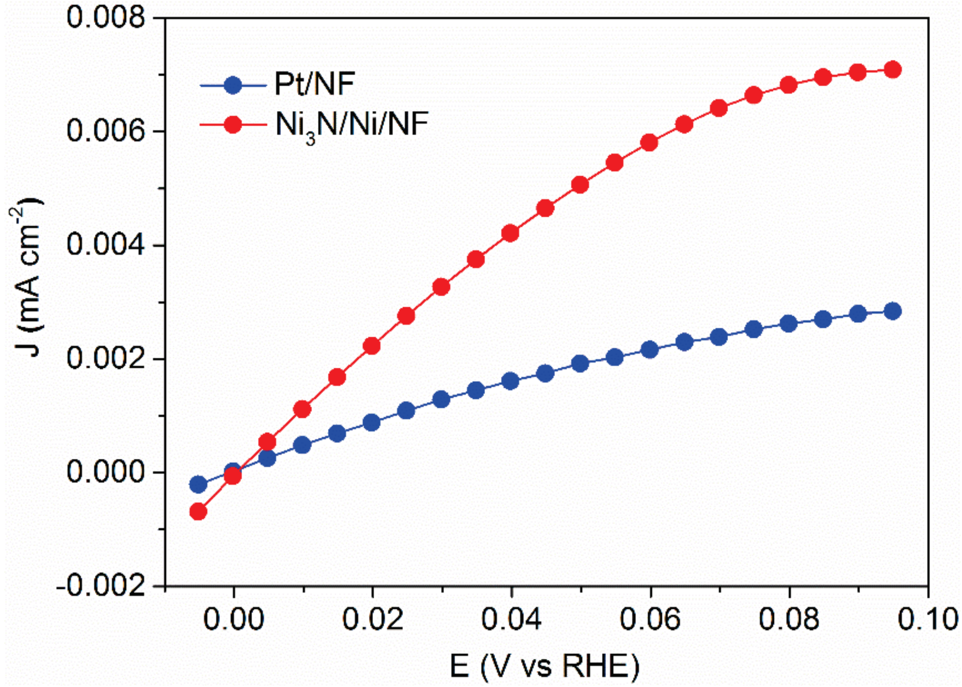

Supplementary Fig. 49. The ECSA-normalized steady-state polarization curves of optimized Pt/NF (1.5 mg/cm^2) and Ni_3N/Ni/NF for HOR in 0.1 M KOH.

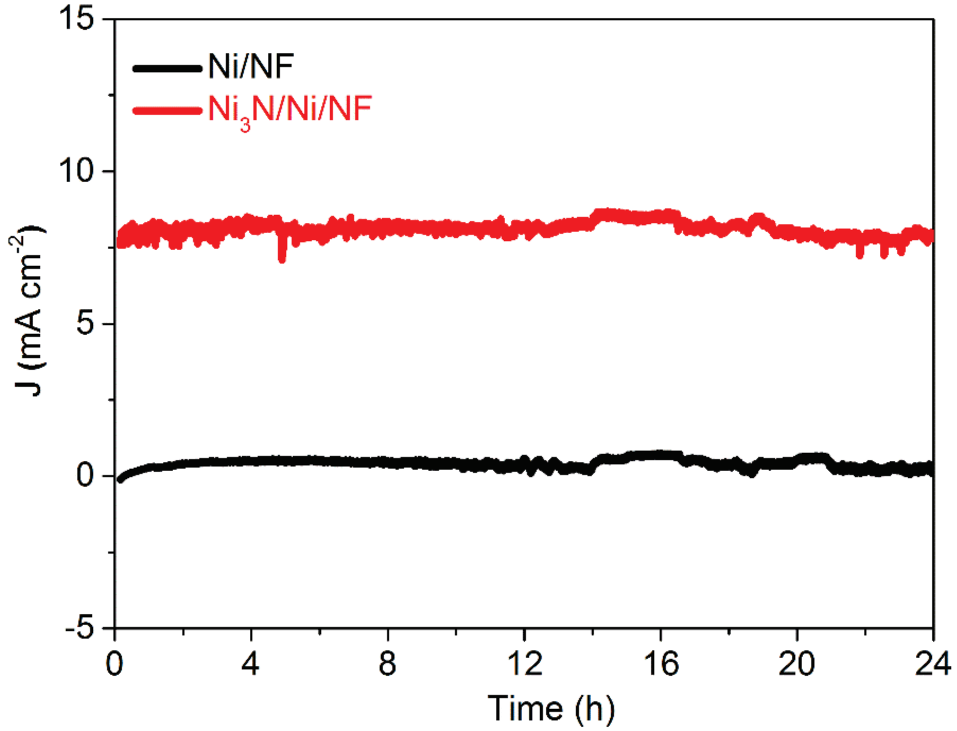

Supplementary Fig. 50. Chronoamperometry curves of Ni_3N/Ni/NF and Ni/NF at 0.09 V vs. RHE in H_2-saturated 0.1 M KOH.

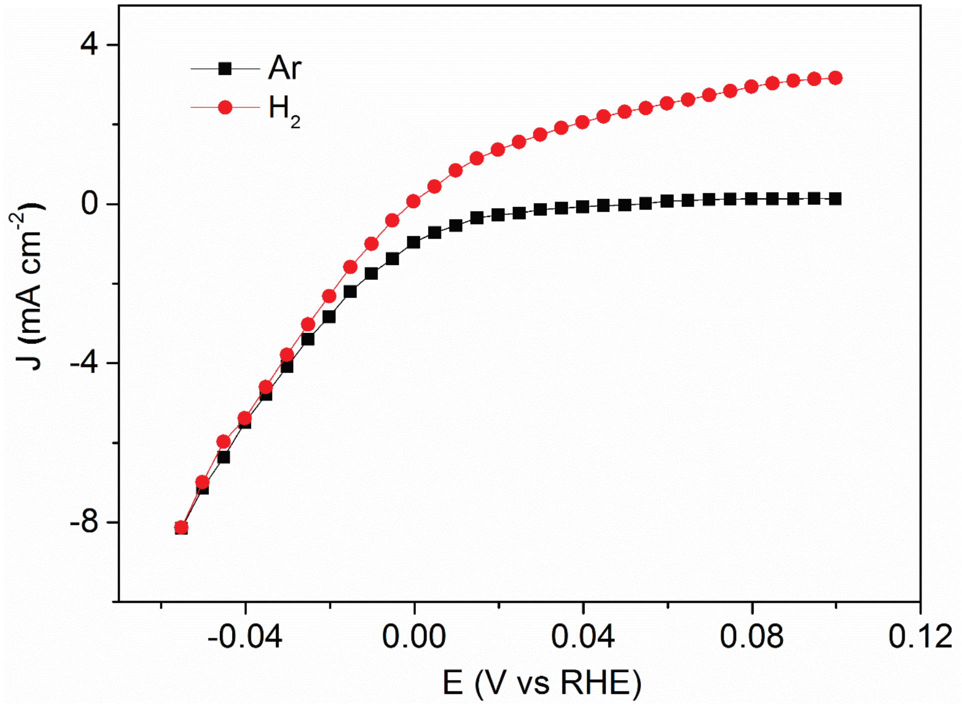

Supplementary Fig. 51. Steady-state polarization curves of Ni_3N/Ni/CF in Ar and H_2-saturated 0.1 M KOH.

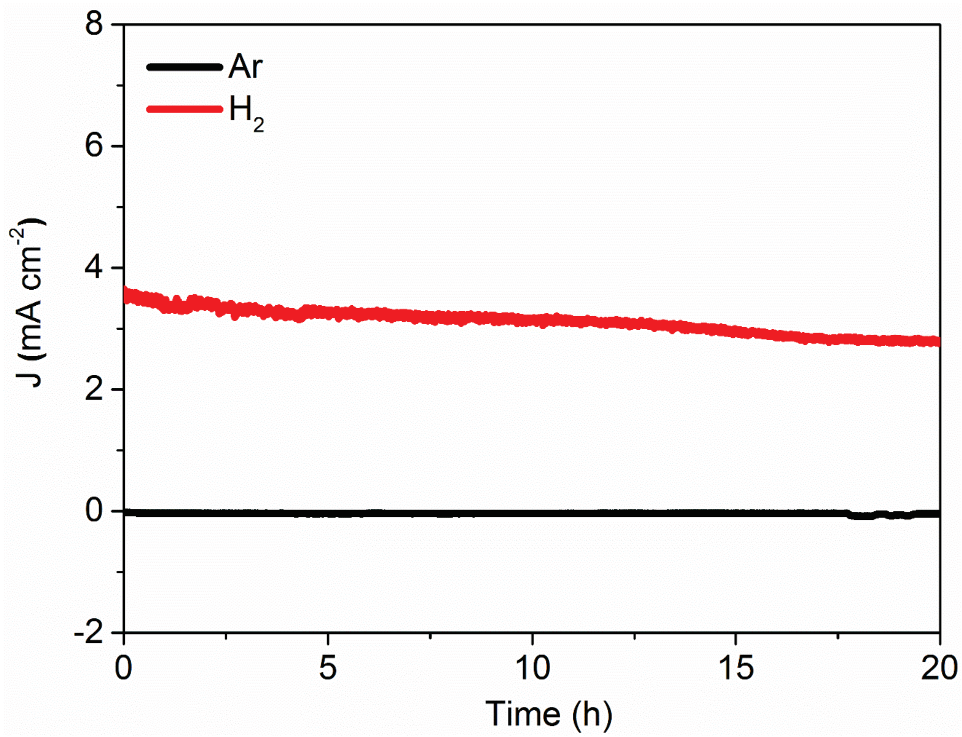

Supplementary Fig. 52. Chronoamperometry curves of Ni_3N/Ni/CF at 0.09 V vs. RHE in Ar and H_2-saturated 0.1 M KOH.

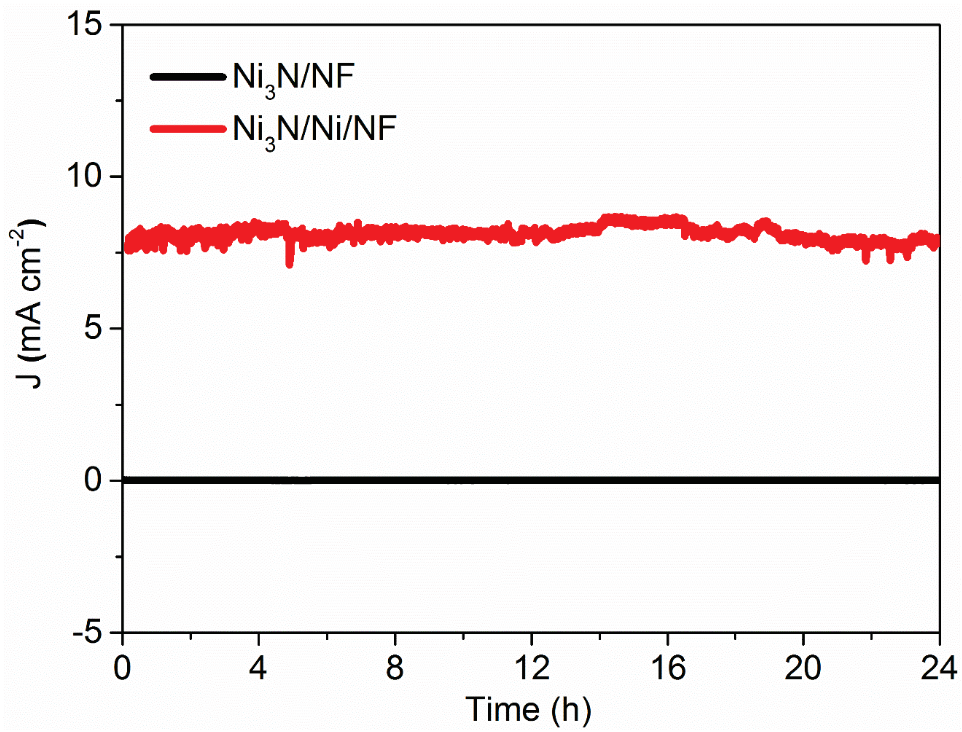

Supplementary Fig. 53. Chronoamperometry curves of Ni_3N/Ni/NF and Ni_3N/NF at 0.09 V vs. RHE in H_2-saturated 0.1 M KOH.

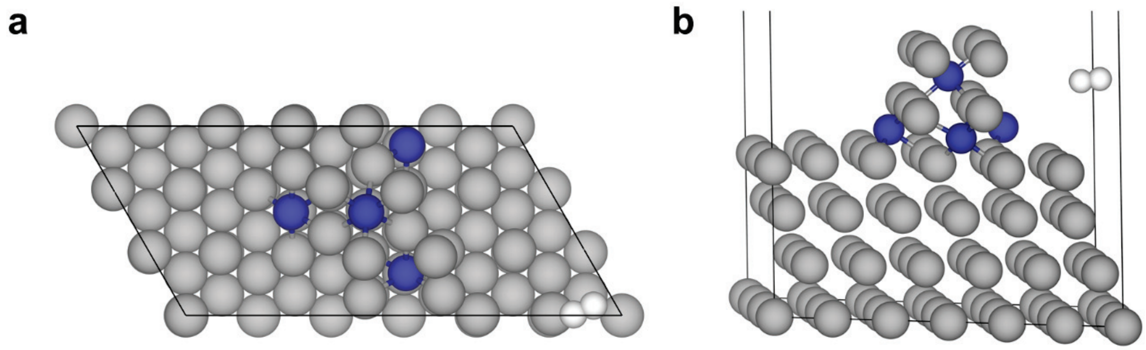

Supplementary Fig. 54. H_2 adsorption structures. a Top view and **b** side view of H_2 adsorption on Ni_3N/Ni. Color code: Ni: grey; N: blue; H: white. Adsorption energy for H_2 adsorption: -0.02 eV.

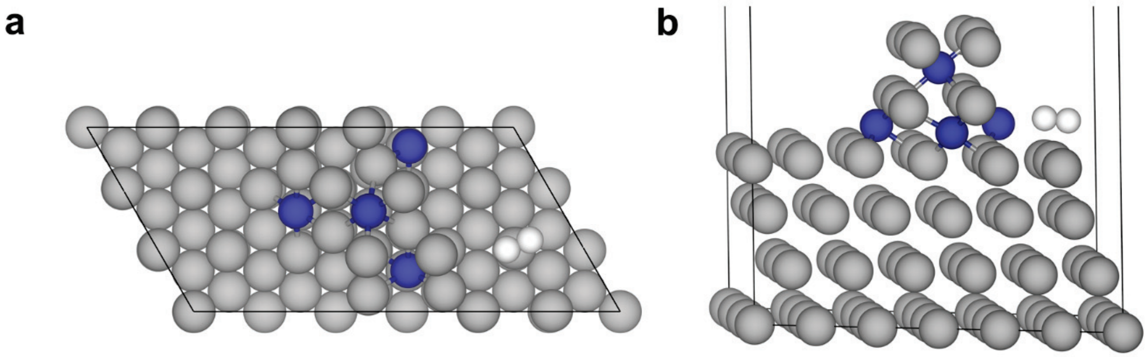

Supplementary Fig. 55. H_2 adsorption structures. a Top view and **b** side view of H_2 adsorption on Ni_3N/Ni. Color code: Ni: grey; N: blue; H: white. Adsorption energy for H_2 adsorption: -0.16 eV.

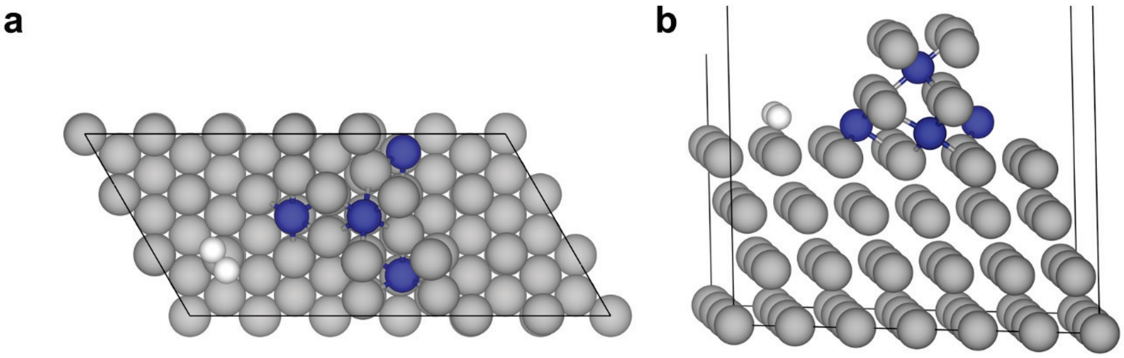

Supplementary Fig. 56. H_2 adsorption structures. a Top view and **b** side view of H_2 adsorption on Ni_3N/Ni. Color code: Ni: grey; N: blue; H: white. Adsorption energy for H_2 adsorption: -0.18 eV.

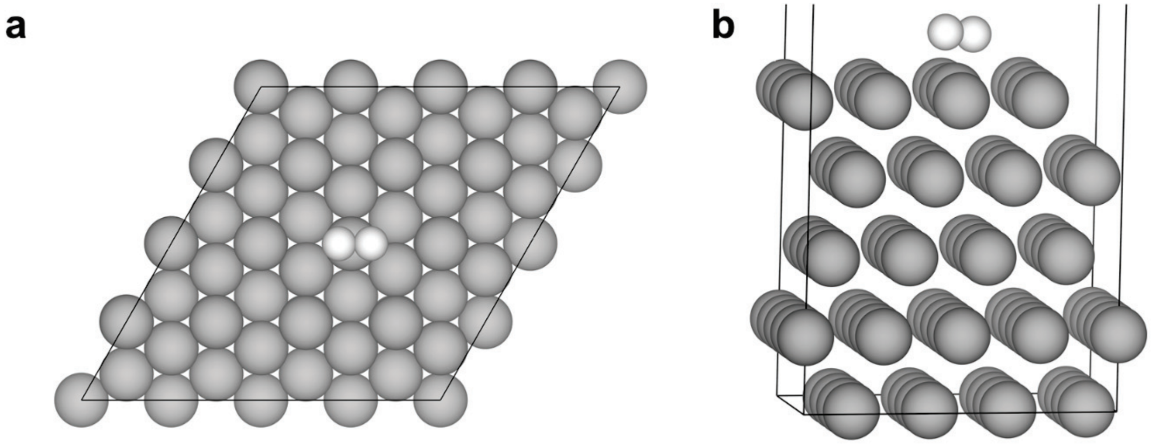

Supplementary Fig. 57. H_2 adsorption structures. a Top view and **b** side view of H_2 adsorption on Ni. Color code: Ni: grey; H: white. Adsorption energy for H_2 adsorption: -0.26 eV.

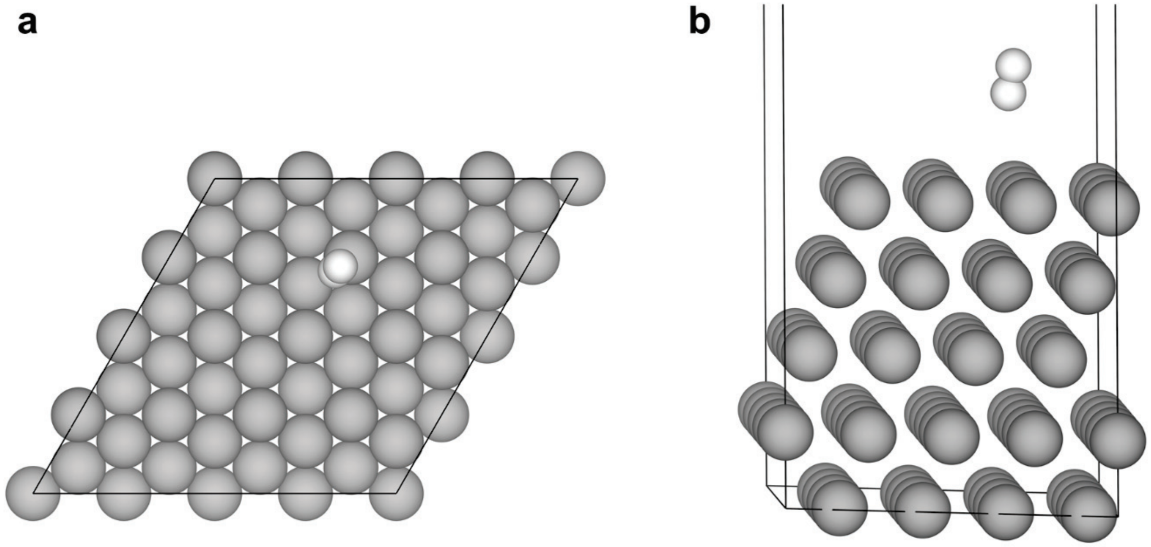

Supplementary Fig. 58. H_2 adsorption structures. a Top view and **b** side view of H_2 adsorption on Ni. Color code: Ni: grey; H: white. Adsorption energy for H_2 adsorption: 0.004 eV.

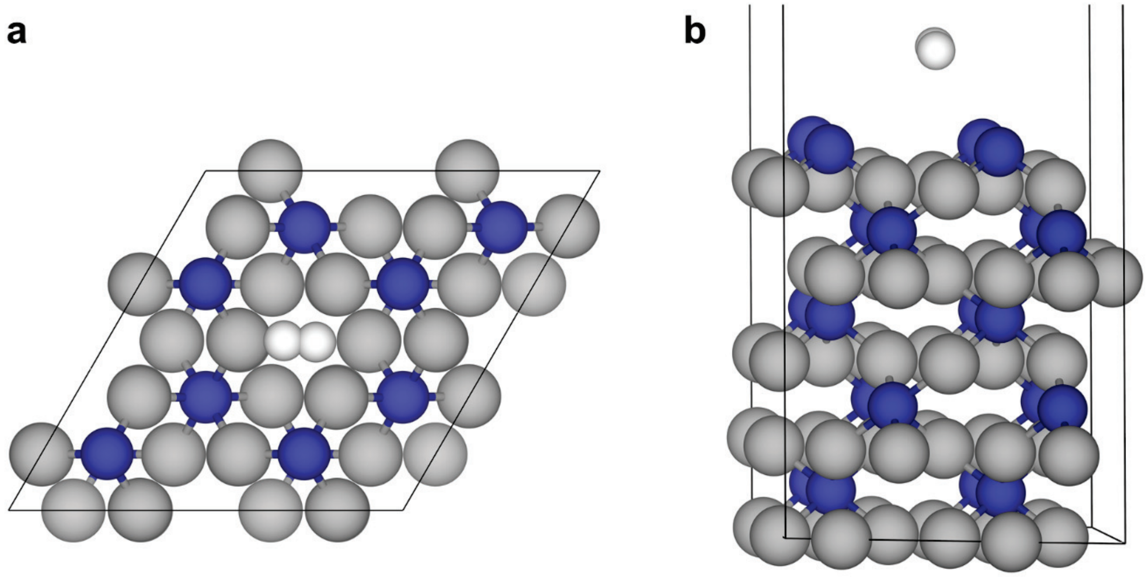

Supplementary Fig. 59. H_2 adsorption structures. a Top view and **b** side view of H_2 adsorption on Ni_3N. Color code: Ni: grey; N: blue; H: white. Adsorption energy for H_2 adsorption: 0.06 eV.

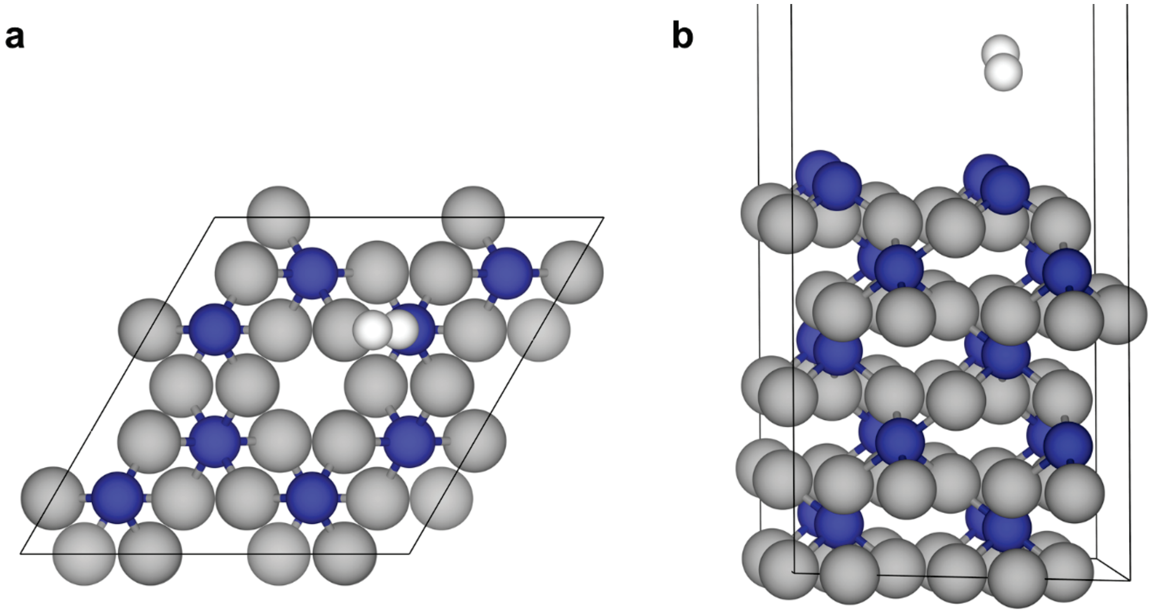

Supplementary Fig. 60. H_2 adsorption structures. a Top view and **b** side view of H_2 adsorption on Ni_3N. Color code: Ni: grey; N: blue; H: white. Adsorption energy for H_2 adsorption: 0.05 eV.

Supplementary Table 1. The specific BET surface areas of Ni_3N/Ni/NF and Pt/NF.

Sample	Specific BET surface area of electrode[a] ($m^2\ g^{-1}$)
Ni_3N/Ni/NF	1.61
Pt/NF (1.5 mg cm^{-2})	6.22
Pt/NF (2.5 mg cm^{-2})	4.47

[a]The specific BET area is calculated on the basis of the total mass of Ni_3N/Ni/NF or Pt/NF.

Supplementary Table 2. The geometric areas and electrochemically active surface areas (ECSAs) of Ni_3N/Ni/NF and Pt/NF.

Sample	Geometric area (cm^2)	ECSA (cm^2)
Ni_3N/Ni/NF	0.25	247
Pt/NF (1.5 mg cm^{-2})	0.25	450
Pt/NF (2.5 mg cm^{-2})	0.25	680

Supplementary Table 3. Comparison of the electrocatalytic performances of Ni_3N/Ni/NF for the HER with those of reported electrocatalysts.

**Electrocatalyst**	η_{10} **(mV)**[a]	η_{100} **(mV)**[b]	**Electrolyte**	**Reference**
Ni_3N/Ni/NF	19	126	1.0 M KPi	**This work**
Ni_3N/Ni/NF	12	64	1.0 M KOH	
Ru@C_2N	17	N/A	1.0 M KOH	*Nat. Nanotech.*, **2017**, 12, 441
Pt/C	20.7	N/A	1.0 M KOH	
Ru/C_3N_4/C	79	N/A	0.1 M KOH	*J. Am. Chem. Soc.*, **2016**, 138, 16174
Ru@N-doped carbon	100	N/A	1.0 M PBS	*Energy Environ. Sci.*, **2018**, 11, 800
	32	N/A	1.0 M KOH	
RuP_2@N,P-doped carbon	57	N/A	1.0 M PBS	*Angew. Chem. Int. Ed.*, **2017**, 56, 11559
	52	N/A	1.0 M KOH	
Pt/C	57	N/A	1.0 M PBS	
	50	N/A	1.0 M KOH	
Cobalt-embedded N-rich carbon nanotubes	540	N/A	0.1 M PBS	*Angew. Chem. Int. Ed.*, **2014**, 53, 4372
	370	N/A	1.0 M KOH	
$MoNi_4$/MoO_2@Ni foam	15	~39	1.0 M KOH	*Nat. Commun.*, **2017**, 8, 15437
FeP nanoparticles	102	N/A	1.0 M PBS	*ACS Nano*, **2014**, 8, 11101
Pt on 2H-WS_2 nanosheets	50	N/A	1.0 M KOH	*Adv. Mater.*, **2018**, 30, 1704779
Ni_3S_2/Ni foam	170	N/A	1.0 M KPi	*J. Am. Chem. Soc.*, **2015**, 137, 14023
	223	N/A	1.0 M KOH	
NiS_2/MoS_2 interface	284	N/A	0.1 M PBS	*ACS Catal.*, **2017**, 7, 6179
	204	N/A	1.0 M KOH	
Mn-doped CoP nanosheets Ti mesh	86	250	0.1 M PBS	*ACS Catal.*, **2017**, 7, 98
	76	150	1.0 M KOH	
Ni_2P embedded in N-doped carbon nanofibers	185.3	~380	1.0 M PBS	*Angew. Chem. Int. Ed.*, **2018**, 57, 1963
	104.2	~200	1.0 M KOH	
$Ni_{0.89}Co_{0.11}Se_2$ nanosheets on Ni foam	82	~310	1.0 M PBS	*Adv. Mater.*, **2017**, 29, 1606521
	85	~160	1.0 M KOH	
Ni-C-N nanosheets	92.1	N/A	1.0 M PBS	*J. Am. Chem. Soc.*, **2016**, 138, 14546
	30.8	~110	1.0 M KOH	
Co-P film on Cu foil	94	158	1.0 M KOH	*Angew. Chem. Int. Ed.*, **2015**, 54, 6251
CoP nanowires on carbon cloth	106	~300	1.0 M PBS	*J. Am. Chem. Soc.*, **2014**, 136, 7587
	209	~520	1.0 M KOH	
MoS_{2+x} on Ni foam	210	335	1.0 M KOH	*Angew. Chem. Int. Ed.*, **2015**, 54, 664
Ni_5P_4 nanosheets on Ni foil	150	N/A	1.0 M KOH	*Angew. Chem. Int. Ed.*, **2015**, 54, 12361
NiSe/Ni foam	96	N/A	1.0 M KOH	*Angew. Chem. Int. Ed.*,

				**2015**, 54, 9351
Co-Ni-P nanowires on Ni foam	52	137	1.0 M KOH	*J. Power Sources*, **2016**, 330, 156.
CoP nanowires on Co foam	124	244	1.0 M KOH	*Chem. Sci.*, **2017**, 8, 2952
Co_3Se_4 nanowires on Co foam	179	262	1.0 M KOH	*Adv. Energy Mater.*, **2017**, 7, 1602579
N-modified Ni	64	203	1.0 M KPi	*J. Am. Chem. Soc.*, **2017**, 139, 12283
Ni_3N nanosheets/carbon microfibers	115	N/A	1.0 M KOH	*J. Mater. Chem. A*, **2017**, 5, 9377
Ni_3N@Ni-Bi/Ti	265	N/A	0.5 M KBi	*J. Mater. Chem. A*, **2017**, 5, 7806
Ni_3N-$Ni(OH)_2$ interface on Ti mesh	~60	181	1.0 M KOH	*J. Mater. Chem. A*, **2018**, 6, 833
Ni_3N nanosheets on carbon cloth	112	~380	1.0 M PBS	*J. Mater. Chem. A*, **2016**, 4, 17363
	305	~470	1.0 M KOH	
TiN@Ni_3N	21	N/A	1.0 M KOH	*J. Mater. Chem. A*, **2016**, 4, 5713
Ni_3N/$Ni(OH)_2$ interface on Ni foam	100	250	1.0 M KOH	*J. Mater. Chem. A*, **2015**, 3, 8171
Ni_3N nanosheets on catbon cloth	136	~350	1.0 M KOH	*Inorg. Chem. Front.*, **2017**, 4, 1120
Ni_3N on Ni foam	121	254	1.0 M KOH	*Electrochim. Acta*, **2016**, 191, 841
Pt-decorated Ni_3N nanosheets	50	120	1.0 M KOH	*Adv. Energy Mater.*, **2017**, 7, 1601390
NiMoN/Ni_3N nanosheets on carbon cloth	31	200	1.0 M KOH	*Nano Energy*, **2018**, 44, 353
Ni_3N/Ni foam	~100	150	1.0 M KOH	*Adv. Energy Mater.*, **2017**, 7, 1601735
Ni_3N-Co	194	~290	1.0 M KOH	*Adv. Mater.*, **2018**, 30, 1705516
Ni_3FeN/reduced graphene oxide	94	~210	1.0 M KOH	*ACS Nano*, **2018**, 12, 245
Fe_2Ni_2N	180	~280	1.0 M KOH	*Inorg. Chem. Front.*, **2016**, 3, 630
Ni_3FeN/carbon cloth	105	~450	1.0 M KOH	*ACS Appl. Mater. Interfaces*, **2018**, 10, 3699

a The overpotential required to deliver -10 mA cm^{-2}. **b** The overpotential required to deliver -100 mA cm^{-2}. The current density is calculated on the basis of the geometric electrode area.

Supplementary Table 4. ZPE values, adsorption energies and free energies for H adsorption.

	frequencies /meV	ΔE /eV	ΔG/eV
Ni	138	-0.54	-0.30
	106		
	103		
Ni_2	139	-0.53	-0.29
	105		
	101		
Ni_3N_N	408	-0.93	-0.57
	92		
	87		
Ni_3N_hollow	130	-0.25	0.01
	128		
	116		
Ni_3N/Ni_N	397	-0.36	0.01
	106		
	102		
Ni_3N/Ni_hollow	129	-0.29	-0.07
	92		
	79		
Ni_3N/Ni_N_2	391	-0.09	0.26
	90		
	78		

Supplementary Methods

Chemicals

All chemicals were used as received without any further purification. Ammonium chloride (NH_4Cl), nickel chloride ($NiCl_2 \cdot 6H_2O$), potassium hydroxide (KOH), potassium phosphate dibasic (K_2HPO_4), potassium phosphate monobasic (KH_2PO_4) and potassium hexafluorophosphate (KPF_6) were purchased from Fisher Chemical. Nickel foam (NF, > 99.99%, 80–110 pores per inch) was purchased from MTI. Sulfuric acid (H_2SO_4), hydrochloric acid (HCl) and anhydrous acetonitrile (CH_3CN) were purchased from Pharmco. Commercial Pt/C (20 wt%) was purchase from Fuel Cell Store. NH_3 gas was obtained from Praxair. Water deionized (18 MΩ·cm) with a Barnstead E-Pure system was used in all experiments.

Characterization

Scanning electron microscopy (SEM) measurement and elemental mapping analysis were conducted on a FEI Quanta 650 FEG microscope equipped with an INCA 350 spectrometer (Oxford Instruments) for energy dispersive X-ray (EDX) spectroscopy. Transmission electron microscopy (TEM) measurement was carried out on a JEM-2800 (JEOL, Japan). X-ray diffraction (XRD) patterns were obtained on a Rigaku MinifexII Desktop X-ray diffractometer. The generated H_2 during electrolysis was detected with a SRI gas chromatograph system 8610C equipped with a HayesSep D packed column, a molecular sieve 13 × packed column, and a thermal conductivity detector. The oven temperature was maintained at 80 °C and argon was used as the carrier gas. The X-ray photoelectron spectroscopy (XPS) analyses were performed using a Kratos Axis Ultra instrument (Chestnut Ridge) at the Surface Analysis Laboratory of Nanofab at the University of Utah. The surface area measurements were performed with N_2 adsorption/desorption isotherms at liquid nitrogen temperature (77 K) using automatted gas sorption analyzer (Quantachrome Instruments) after dehydration under vacuum at 423 K for 12 h

Syntheses of electrocatalysts

Synthesis of Ni/carbon foam (Ni/CF) and Ni_3N/Ni/carbon foam (Ni_3N/Ni/CF): The Ni_3N/Ni/CF electrodes were prepared by cathodic electrodeposition of Ni particles on carbon foams followed by thermal nitridation. The electrodeposition was carried out with a two-electrode configuration in a cell containing 2.0 M NH_4Cl and 0.1 M $NiCl_2$ at room temperature. A piece of clean carbon foam (0.5 cm × 0.5 cm) and a carbon rod were used as the working and counter electrodes, respectively. The electrodeposition was performed at a constant current density of −1.0 A cm^{-2} for 500 s under N_2 protection without stirring to obtain Ni/CF. Then the resultant Ni/CF was placed in the center of a quartz tube purged with NH_3 flow. It was heated to 300 °C at a ramping rate of 10 °C min^{-1} and maintained at the same temperature for 6 h. Finally, the furnace was naturally cooled down to room temperature, leading to Ni_3N/Ni/CF. The NH_3 flow was kept throughout the whole process.

Synthesis of Ni_3N/nickel foam (Ni_3N/NF): A piece of clean nickel foam (0.5 cm × 0.5 cm) was directly placed in the center of a quartz tube purged with NH_3 flow. It was heated to 300 °C at a ramping rate of 10 °C min^{-1} and maintained at the same temperature for 6 h. Finally, the furnace was naturally cooled down to room temperature, leading to Ni_3N/CF. The NH_3 flow was kept throughout the whole process.

Electrochemically active surface areas measurement

The electrochemically active surface areas (ECSA) of these electrodes were determined according to an established methodology reported in the literature (*J. Am. Chem. Soc.* **2018**, 140, 2397). In order to avoid the ion transfer reactions at the electrode interface, non-aqueous electrochemical double layer capacitance measurements were conducted using a pseudo-reference electrode consisting of Ag/AgCl wire bathed in 0.15 M KPF_6/CH_3CN solution and separated from solution by a Vycor frit. Specifically, cyclic voltammetry curves of the electrodes were collected over a narrow range (±50 mV) centered around the open circuit potential (OCP) in 0.15 M KPF_6 with CH_3CN electrolyte. CV cycling was repeated using a range of scan rates from 4 to 20 mV s^{-1}. The electrochemical double-layer capacitance (C_{dl}) was then estimated by plotting the difference between the anodic and cathodic current densities ($\Delta j = j_a - j_c$) at OCP against the scan rate. The resulting linear slope is twice of the C_{dl}. The ESCA can be calculated according to the following equation: ECSA = C_{dl} /C_s, where C_s is the specific capacitance of a flat smooth surface of the electrode material, which is assumed to be 11 μF cm^{-2} according to the literature report (*J. Am. Chem. Soc.* **2018**, 140, 2397)

Free energy calculations

The Gibbs free energies were obtained by including zero-point energy (ZPE) and entropy corrections.

$$\Delta G = \Delta E + (ZPE - T\Delta S) \qquad (1)$$

The ZPE were calculated by summing over vibrational modes from frequency calculations.

$$ZPE = \sum_{i}^{modes} \frac{1}{2} h\upsilon_i \qquad (2)$$

TS for H_2 gas at 298 K is equal to -0.202 eV. [NIST Chemistry WebBook, https://webbook.nist.gov/cgi/cbook.cgi?ID=C1333740&Mask=1] The TS for adsorbed species is set to zero.
